# Parameter-free optimization algorithm effective and precise solution of the optimal power flow problem

**DOI:** 10.1038/s41598-026-48197-9

**Published:** 2026-05-15

**Authors:** Saket Gupta, Neeraj Kumar, Mohd Zuhaib, Marwan Ahmad Abdullah Sufyan, Upma Singh, Sandeep Sharma, Vijay Bhuria, Ankita Gupta

**Affiliations:** 1 Instrumentation and Control Engineering Department, BVCOE, New Delhi, 110063 India; 2 Electrical and Electronics Engineering Department, BVCOE, New Delhi, 110063 India; 3 Department of Electrical Engineering, GLA University, Mathura, 281406 India; 4 College of Engineering and Information Technology, Aljanad University of Science and Technology, Taiz, Yemen; 5 Department of Electronics and Communication Engineering, MSIT, Delhi, 110058 India; 6 Department of Electrical Engineering, Madhav Institute of Technology & Science, Deemed University, Gwalior, India; 7 Department of Artificial Intelligence and Machine Learning, ADGITM, Delhi, 110053 India

**Keywords:** Artificial intelligence, Jaya, Nature-inspired algorithms, OPF, Energy science and technology, Engineering, Mathematics and computing

## Abstract

Difficult Optimization problems can be efficiently solved through nature-inspired optimization algorithms, which remain problem-independent and are computational models at a conceptual level. In this work, a Jaya algorithm will be offered for tackling the Optimal Power Flow (OPF) problem. The basic idea of the Jaya algorithm for optimization problems is that the candidate solutions should move closer to the global optimum solution without converging at suboptimal solutions. Like other nature-inspired optimization techniques, the Jaya method is parameter-free and does not require any algorithm-specific control factors, such as learning or mutant parameters. The parameter freedom of an optimization algorithm not only improves its simplicity, but it also overcomes the challenge of adjusting optimization algorithm parameters, which affects performance as indicated in the literature, and can be expensive for some computations. The OPF problem aims to optimize the different objective functions using the control variables of the power systems. These include improving the voltage stability, decreasing the cost and emissions, and reducing power loss. For comparison purposes, the Jaya optimization technique for the OPF problem has been tested on the IEEE 30-Bus, 57-Bus and IEEE 118 Bus systems. The results were compared to previously reported outcomes of optimization approaches. The result of the computational study reveals the effectiveness of the Jaya optimization technique when compared with the other reported techniques. For instance, when the Jaya algorithm was applied to the IEEE 30-bus system, the fuel cost was reduced by approximately 11.31% compared to the initial operating condition.

## Introduction

Artificial Intelligence (AI) can be considered an interdisciplinary field of study, which has different perspectives for the development of Intelligent Systems. These systems have the ability to perform different functions, which are typically done by human beings, such as reasoning, solving problems, learning, perceiving, and comprehending languages. The Nature-inspired (NI) algorithms are part of artificial intelligence. NI algorithms, which are metaheuristic algorithms based on processes observed in nature, are frequently used in AI systems to improve performance, solve complex optimization tasks, and simulate intelligent behavior.

Over the previous two decades, the globe has seen a population increase as well as enormous technical advancement. All of this has caused a rise in global energy consumption. The ubiquitous application of OPF solutions spans the operation, control, planning, and even future expansion of electrical power systems^1^. Within a network, OPF algorithms employ control variables within feasible regions to optimize predefined objectives. Minimizing fuel cost is a frequent primary objective, but others like voltage stability enhancement, voltage profile improvement, loss reduction, and emission minimization can also be included through adjustments to continuous and discrete control variables. Common control variables for OPF include generator real power outputs (except the slack bus), bus voltages, transformer tap settings, phase shifters, shunt capacitors, and FACTS controllers. Mathematically, OPF exhibits a complex structure characterized by nonlinearity, high dimensionality, non-differentiability, multi-modality, non-convexity, and a mix of discrete and continuous control variables. At the beginning of 60’s, Carpentier addressed the OPF problem as an extension of economic load dispatch for the first time in history^2^.

Traditional approaches significantly focused on solving the OPF problem through classical methods^3^. These attempts often took advantage of the gradients or derivatives of the objective function to search for an optimal solution, but were often difficult to execute because these approaches had a tendency to get stuck in local optima instead of true global minimum. Furthermore, their range of success was greatly reduced due to problems such as non-convexity, non-differentiability, multi-modality, and dependence on starting points. These problems made classical methods highly inefficient when dealing with the challenges presented by OPF.

A large variety of complex optimization problems have been successfully resolved with NI algorithms. NI algorithms are, however, unconstraint search techniques and require clear mechanisms to continue searching in restricted areas. Therefore, constraint optimization problem is of great importance within the optimization field because, due to their physical, geometrical and other limitation, most problems with optimization have different types of constraints. The method of handling constraints plays a very important role when using Nature-inspired algorithms to solve constraint optimization problems.

A large variety of complex optimization problems have been successfully resolved with Nature-inspired algorithms. The NI algorithms are not based on the structures mathematics does, meaning they perform perfectly in conditions of non-differentiability, non-smoothness, multi-objectivity, and non-convexity. This kind of flexibility, along with swift improvements in branches of Nature-inspired algorithms, made it attractive to apply these techniques to OPF^4^ and other complex optimization problems. The power engineers have been fascinated in the past few years with the hundreds of successful applications using NI algorithms to solve different optimization problems, OPF being one of them. Hybrid Rao-2 sine cosine algorithm^5^, Improved Liver Cancer Algorithm^6^,, improved pelican optimization algorithm^7^, hybrid artificial rabbits’ optimization^8^, Spider Wasp Optimization Algorithm^9^ Black-Hole Optimization^10^, Linear Adaptive Genetic Algorithm^11^, MSA^12^, WEA^13^, SOSA^14^, Tree-seed^15^, DSA^16^ have demonstrated notable results. A concise review of selected relevant studies is summarized in Table 1.

**Table 1:** A comparative review of selected relevant works.

S. no	Year	Paper title	OPF type/system	Optimization/solution technique	Main contribution
1	2025 ^1^	Solving Single- and Multi-Objective Optimal Power Flow Problems Using the Spider Wasp Optimization Algorithm	Single & Multi-objective AC OPF	Spider Wasp Optimization Algorithm (SWOA)	Provides efficient solutions for both single and multi-objective OPF problems with improved convergence accuracy and stability.
2	2025 ^5^	Optimal Power Flow Solutions for Normal and Critical Loading Scenarios Using Hybrid Rao-2 Sine Cosine Algorithm	AC OPF under normal and critical loading	Hybrid Rao-2 Algorithm + Sine Cosine Algorithm	Demonstrates improved convergence, stability, and solution quality for OPF under both normal and stressed loading conditions, outperforming conventional methods.
3	2024 ^6^	Stochastic Optimal Power Flow Framework with Incorporation of Wind Turbines and Solar PVs Using an Improved Liver Cancer Algorithm	Stochastic OPF with Wind & Solar PV	Improved Liver Cancer Algorithm (ILCA)	Effectively manages renewable uncertainty and improves fuel cost, power loss, and voltage stability performance.
4	2025^7^	An Improved Pelican Optimization Algorithm for Solving Stochastic Optimal Power Flow Problem Considering Renewable Energy Uncertainty	Stochastic OPF with RES	Improved Pelican Optimization Algorithm (POA)	Achieves reliable stochastic OPF solutions under renewable uncertainty with enhanced convergence behavior.
5	2024 ^8^	Optimizing Reactive Power Dispatch in Electrical Networks Using a Hybrid Artificial Rabbits and Gradient-Based Optimization	Reactive Power Dispatch OPF	Hybrid Artificial Rabbits Algorithm + Gradient-Based Optimization	Enhances voltage profile and minimizes power losses through effective reactive power optimization.
6	2025 ^17^	Hybrid Brown-Bear and Hippopotamus Optimization with Quasi-Opposition-Based Learning for Optimal Power Flow with Renewable Energy Integration	AC OPF with RES	Hybrid Brown-Bear Optimization + Hippopotamus Optimization + QOBL	Improves convergence speed and avoids local minima in renewable-integrated OPF while reducing fuel cost, losses, and voltage deviation.
7	2025 ^18^	A generic optimal power flow solution associated with technical improvements and emission reduction by multi-objective ARO algorithm	Multi-objective AC OPF	Arithmetic Optimization Algorithm (ARO)	Simultaneously minimises fuel cost, emission, and technical indices using multi-objective ARO.
8	2025 ^19^	Optimal power flow solution using novel optimization technique: a case study	AC OPF	Novel metaheuristic optimization technique	Demonstrates the effectiveness of a newly proposed optimization technique for improving OPF solution quality and convergence through a practical case study.
9	2024 ^20^	A Modified Artificial Hummingbird Algorithm for Solving Optimal Power Flow Problem in Power Systems	AC OPF	Modified Artificial Hummingbird Algorithm (MAHA)	Introduces MAHA to enhance convergence speed and solution quality for OPF, outperforming classical metaheuristics on standard IEEE test systems.
10	2024 ^21^	Chaotic-Quasi-Oppositional-Phasor Based Multi-Populations Gorilla Troop Optimizer for Optimal Power Flow Solution	AC OPF	Chaotic-Quasi-Oppositional Gorilla Troop Optimizer (CQO-GTO)	Applies CQO-GTO to improve exploration and exploitation for OPF, achieving better convergence and robustness for multi-objective power system optimization.
11	2023 ^22^	Optimal power flow solution using a learning-based sine–cosine algorithm	AC OPF	Learning-Based Sine Cosine Algorithm (LB-SCA)	Enhances conventional SCA by incorporating learning mechanisms to improve convergence speed, solution accuracy, and robustness for OPF problems.
12	2023 ^23^	Gaussian bare-bones levy circulatory system-based optimization for power flow in the presence of renewable units	Renewable-integrated AC OPF	Gaussian Bare-Bones Levy Circulatory System Optimization	Enhances exploration–exploitation balance and robustness of OPF solutions under renewable uncertainty using Gaussian distribution and Levy flight mechanisms.
13	2014 ^24^	Multi-objective optimal power flow considering the cost, emission, voltage deviation and power losses using multi-objective modified imperialist competitive algorithm	Multi-objective AC OPF	Multi-Objective Modified Imperialist Competitive Algorithm (MOMICA)	Simultaneously minimizes fuel cost, emissions, voltage deviation, and active power losses, establishing a benchmark framework for multi-objective OPF optimization.
14	2025 ^25^	Optimal power flow in power systems with renewable energy resources uncertainty, including geothermal power plants	Probabilistic OPF with RES & geothermal	Probabilistic modeling + metaheuristic optimization	Incorporates geothermal power plants along with wind and solar uncertainties to improve the feasibility and reliability of OPF solutions.
15	2022 ^26^	Solution of probabilistic optimal power flow incorporating renewable energy uncertainty using a novel circle search algorithm	Probabilistic OPF with RES	Circle Search Algorithm	Proposes a novel circle search optimizer to efficiently handle renewable uncertainty in probabilistic OPF while minimizing cost and losses.

NI algorithms are random search techniques that are simple to formulate, more convenient compared to analytical methods, and efficient in identifying solutions very close to optimal values. On the flip side, there are chances of fast convergence of the techniques in case of improper selection of parameters of the algorithm; therefore, in order to overcome poor/non-optimal solutions, there is a need for optimization of the NI algorithms’ parameters. Parameter selection of NI techniques is an arduous task and time-consuming as well. In overcoming the said drawback, the authors of the current research present parameter-less optimization techniques of the algorithm, called ‘Jaya Algorithms’. This feature of having “no dependency of exploration and exploitation search capability of the algorithm on the specific factors of the algorithm” makes the search capability of the current approach better compared to the previous studies mentioned in Section I.

However, one key question emerges: What is the point of creating new algorithms when there are a multitude of existing ones? The solution lies in the inherent trade-offs present in each of the NI algorithms. They attempt to maintain a balance between the two actions: exploration, searching new areas, and exploitation, optimizing the promising solutions. Some algorithms perform well in the former but not in the latter, and the reverse, too. Moreover, each algorithm has its own set of pros and cons, depending on the nature of the problem it tackles. This is backed by the “No Free Lunch” theorem, which states that there is no optimal algorithm for broad optimization tasks^27^.In order to solve OPF problems, the Jaya method is presented in this study.

The contributions of this paper are:

- The utilization of the parameter-free optimization algorithm, known as the Jaya optimization algorithm, to solve single-objective and multi-objective optimal power flow problems in power systems.
- The implementation and testing of the optimization algorithm on typical IEEE test cases, including large-scale networks like the IEEE 118-bus network.
- The evaluation of the optimization algorithm in terms of solution reliability by running the optimization algorithm several times.
- The comparison with some widely used optimization algorithms shows the strength of the Jaya optimization algorithm in terms of convergence characteristics.

The authors of this article present an application of the Jaya algorithm to solve the OPF problem while accounting for technical and economic objective functions. The suggested algorithm is a parameter less constraint optimization algorithm, so algorithm-specific parameter tuning is not required. This quality makes the algorithm very simple to use and applies to different kinds of optimization problems. The algorithm-specific parameters play a very important role during the search process, and it is a very challenging task for the given problem.

While the proposed implementation of the Jaya algorithm has demonstrated competitive performance across standard IEEE test systems, several limitations must be acknowledged. In particular, since the Jaya is mainly based on the greedy update strategy of Jaya, the risk of premature convergence may arise. To alleviate this issue, three mitigation strategies are employed: (i) random population initialization within sufficiently wide variable bounds, (ii) execution of multiple independent simulation runs, and (iii) incorporation of stochastic terms in the update equations to implicitly enhance exploration capability.

The remainder of the paper is organized like this. Section 2 describes OPF problem modelling. Details on the proposed Jaya algorithm for solving the OPF problem are provided in Sect. 3. The numerical results achieved by the proposed method are given in Sect. 4. In the last section, the conclusion is presented.

## Problem formulation

The OPF problem can be mathematically formulated as follows^28^:

1 $$\:\mathrm{O}\mathrm{p}\mathrm{t}\mathrm{i}\mathrm{m}\mathrm{i}\mathrm{z}\mathrm{e}\:M\:\left(U,\:X\right)$$

Subject to the constraints;

2 $$\:\left\{\begin{array}{c}g\:(U,\:X)\:=\:0\:\:\\\:h\:(U,\:X)\:\le\:\:0\end{array}\right.\:$$

where$\:M\:\left(U,\:X\right)$ indicates the objective function that has to be minimised.

The set of dependent variables is represented by ‘U’ and ‘X’, and it consists of:

Here, *U* can be expressed as

3 $$\:{\mathrm{U}}^{\mathrm{T}}=[{{P}_{{g}_{1}},\mathrm{V}}_{1}\dots\:{\mathrm{V}}_{\mathrm{N}\mathrm{L}\mathrm{B}},{\mathrm{Q}}_{\mathrm{g}1},\dots\:{\mathrm{Q}}_{\mathrm{g}\mathrm{N}\mathrm{G}},{S}_{1},\dots\:{S}_{\mathrm{n}\mathrm{t}\mathrm{l}}]\:\:$$

while *X* can be expressed as

4 $$\:{\mathrm{X}}^{\mathrm{T}}=[{\mathrm{V}}_{{\mathrm{g}}_{1}}\dots\:{\mathrm{V}}_{{\mathrm{g}}_{\mathrm{N}\mathrm{G}\mathrm{N}}},{\mathrm{Q}}_{{\mathrm{C}}_{1}}\dots\:{\mathrm{Q}}_{{\mathrm{C}}_{\mathrm{N}\mathrm{C}}},{\mathrm{T}}_{1}\dots\:{\mathrm{T}}_{\mathrm{N}\mathrm{T}\mathrm{R}}]\:\:\:$$

### Constraints

- Equality Constraints (g):
  Power flow equations are used to define the equality restrictions. Power flow equations are separated into two types: active and reactive. The active and reactive power flow equations are as follows.
  5 $$\:{\:\:\:\:\:\:\:\:\:\:\:\:\:\:\:\:\:\:\:\:0\:\:=\:\:\:\:\:\:\:\mathrm{P}}_{\mathrm{g}\mathrm{i}}-{\mathrm{P}}_{\mathrm{d}\mathrm{i}}-{\mathrm{P}}_{\mathrm{L}\mathrm{o}\mathrm{s}\mathrm{s}}$$
  6 $$\:{\:\:0\:\:=\:\:\:\:\:\:\mathrm{Q}}_{\mathrm{g}\mathrm{i}}-{\mathrm{Q}}_{\mathrm{d}\mathrm{i}}-\:\:\:{\mathrm{Q}}_{\mathrm{L}\mathrm{o}\mathrm{s}\mathrm{s}}\:\:$$
- Inequality Constraints (h):

Power systems are made up of numerous components and equipment. There are limitations to how these devices can operate. These devices’ minimum and maximum limits are known as inequality constraints.

- *Generators constraints*:
  7 $$\:{\:\:\:\:\:\:\:\:\:\:\mathrm{V}}_{{\mathrm{g}}_{\mathrm{k}}}^{\mathrm{m}\mathrm{i}\mathrm{n}}\le\:{\mathrm{V}}_{{\mathrm{g}}_{\mathrm{k}}}\le\:{\mathrm{V}}_{{\mathrm{g}}_{\mathrm{k}}}^{\mathrm{m}\mathrm{a}\mathrm{x}}\:\:\:\:\:\:\:\:\mathrm{k}=1\dots\:...\mathrm{N}\mathrm{G}\mathrm{N}\:\:\:\:\:\:\:\:\:$$
  8 $$\:{\:\:\:\:\:\:\:\:\:\:\mathrm{Q}}_{{\mathrm{g}}_{\mathrm{k}}}^{\mathrm{m}\mathrm{i}\mathrm{n}}\le\:\:{\mathrm{Q}}_{{\mathrm{g}}_{\mathrm{k}}}\le\:{\mathrm{Q}}_{{\mathrm{g}}_{\mathrm{k}}}^{\mathrm{m}\mathrm{a}\mathrm{x}}\:\:\:\:\:\mathrm{k}=1\dots\:\dots\:\mathrm{N}\mathrm{G}\mathrm{N}\:\:\:\:\:\:$$
- *Shunt VAR compensator constraints*:
  9 $$\:{\:\:\:\:\:\:\:\mathrm{Q}}_{{\mathrm{C}}_{\mathrm{k}}}^{\mathrm{m}\mathrm{i}\mathrm{n}}\le\:{\mathrm{Q}}_{{\mathrm{C}}_{\mathrm{k}}}\le\:{\mathrm{Q}}_{{\mathrm{C}}_{\mathrm{k}}}^{\mathrm{m}\mathrm{a}\mathrm{x}}\:\:\:\mathrm{k}=1\dots\:\dots\:\mathrm{N}\mathrm{G}\mathrm{N}\:\:\:\:$$
- *Transformer Constraints*:
  10 $$\:{\:\:\:\:\:\:\:\:\:\:\mathrm{T}}_{\mathrm{k}}^{\mathrm{m}\mathrm{i}\mathrm{n}}\:\le\:{\mathrm{T}}_{\mathrm{k}}\le\:{\mathrm{T}}_{\mathrm{k}}^{\mathrm{m}\mathrm{a}\mathrm{x}}\:\:\:\mathrm{k}=1\dots\:\dots\:\mathrm{N}\mathrm{T}\mathrm{R}\:\:\:$$
- *Security Constraints*:
  11 $$\:{\:\:\:\:\:\:\:\:\:\:\:\:\mathrm{V}}_{{\mathrm{L}}_{\mathrm{k}}}^{\mathrm{m}\mathrm{i}\mathrm{n}}\le\:{\mathrm{V}}_{{\mathrm{L}}_{\mathrm{k}}}\le\:{\mathrm{V}}_{{\mathrm{L}}_{\mathrm{k}}}^{\mathrm{m}\mathrm{a}\mathrm{x}}\:\:\:\:\:\:\:\:\mathrm{k}=1\dots\:\dots\:\mathrm{N}\mathrm{L}\mathrm{B}\:\:\:\:\:\:\:\:$$
  12 $$\:{\:\:\:\:\:\mathrm{S}}_{{\mathrm{l}}_{\mathrm{k}}}\le\:{\mathrm{S}}_{{\mathrm{l}}_{\mathrm{k}}}^{\mathrm{m}\mathrm{a}\mathrm{x}}\:\:\:\:\:\:\:\:\:\mathrm{k}=1\dots\:\dots\:\mathrm{n}\mathrm{t}\mathrm{l}\:\:$$
  Where, P_gi_ &Q_gi_: Generator active and reactive power output of *i*^*th*^bus, P_di_ &Q_di_: Active and reactive power demands at *i*^*th*^bus,$\:{\mathrm{Q}}_{{\mathrm{C}}_{\mathrm{k}}}$=Limits on output of shunt VAR compensators at *k*^*th*^bus, V_Lk_: Load bus voltage limits at *k*^*th*^ bus, $\:{\:\mathrm{S}}_{{\mathrm{l}}_{\mathrm{k}}}$=Line flow limit in MVA, NTR: No of regulating transformer, NGN, and NLB: No of generator and Load bus respectively.

### Incorporation of constraints

Operating constraints, encompassing generator capabilities, VAR limitations, transformer capacities, line flow limits, and bus voltage bounds, are effectively managed through penalty factors within the OPF framework. The objective function has been augmented with penalty terms corresponding to each constraint violation. The mathematical formulations of the penalty functions, along with the selected penalty coefficients and their role in guiding the optimization process toward feasible solutions, have now been clearly presented in Eq. (13).

13 $$\begin{aligned}{\mathrm{M}}_{\mathrm{a}\mathrm{u}\mathrm{g}} & =\mathrm{M}\left(\mathrm{U},\mathrm{X}\right)+{P}_{1}{\sum\:}_{k=1}^{NGN}h\left({P}_{{g}_{Slack}}-{P}_{{g}_{Slack}}^{lim}\right)+{CP}_{2}{\sum\:}_{k=1}^{NGN}h\left({Q}_{{g}_{k}}-{Q}_{{g}_{k}}^{lim}\right) \\ & \quad +{P}_{3}{\sum\:}_{k=1}^{NLB}h\left({V}_{{L}_{k}}-{V}_{{L}_{k}}^{lim}\right)+{P}_{4}{\sum\:}_{k=1}^{NGN}h\left({S}_{{l}_{k}}-{S}_{{l}_{k}}^{lim}\right)\end{aligned}$$

Where$\:{\:\mathrm{P}}_{1}$,$\:{\mathrm{P}}_{2}$, $\:{\mathrm{P}}_{3}$ and $\:{\mathrm{P}}_{4}$ represent penalty factors for limit violations, which are chosen by the user.

## Jaya algorithm

**Fig. 1:**
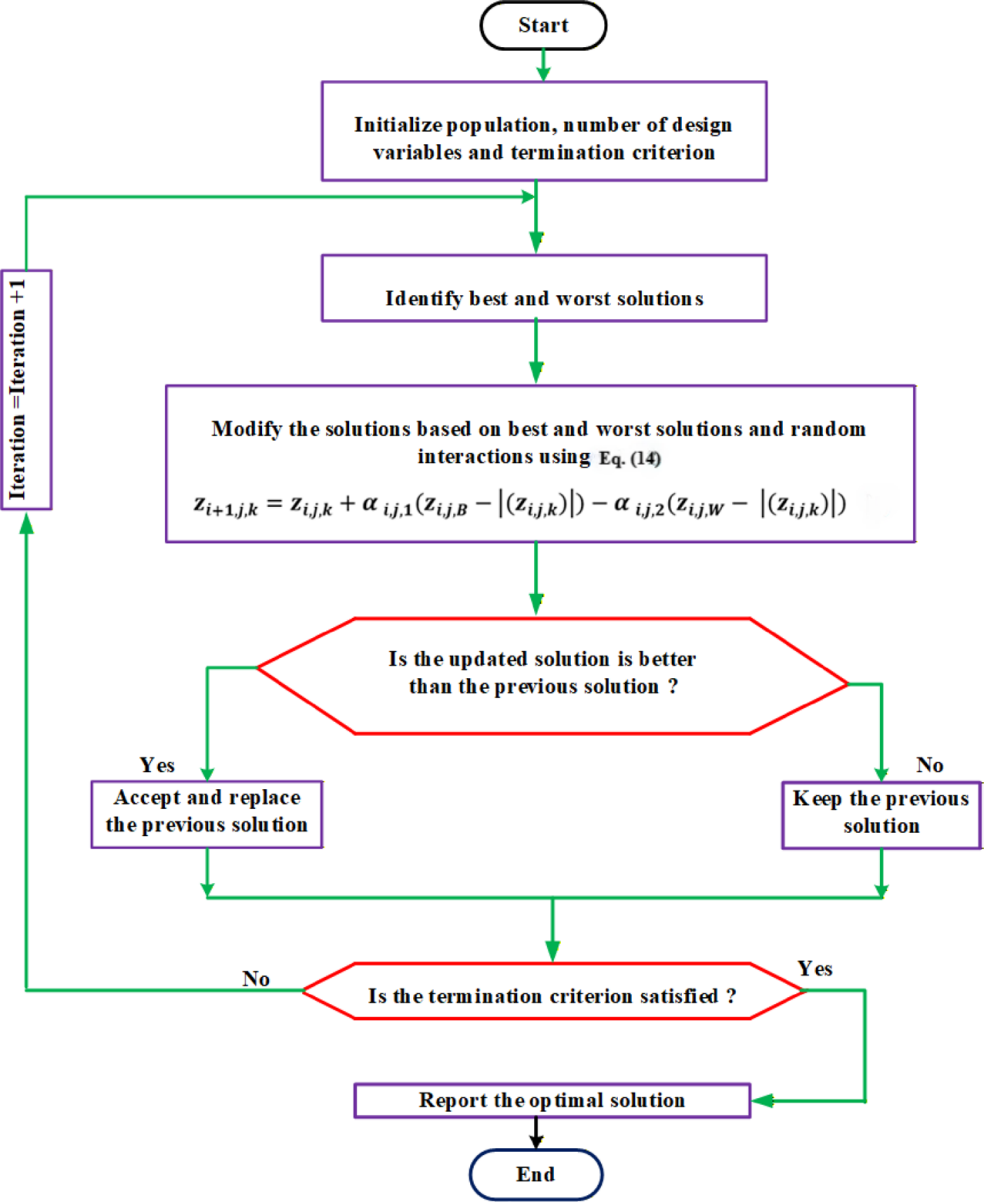
Flowchart for Jaya algorithm.

The Jaya algorithm proposed by Rao^29^ is a powerful global optimization method, which is mainly characterized by the absence of algorithm-specific parameters. This algorithm imitates the natural phenomenon of problem-solving in which every attempt aims toward an optimal solution while avoiding a lesser solution. In this respect, the present algorithm is similar to TLBO, and its non-availability of algorithm-specific parameters makes it easier to implement for various engineering optimization problems. The flow chart of the Jaya algorithm is given in Fig. 1.

***Key characteristics***:

- Objective function: Maximizes (or minimizes) *M(z*).
- Initial solution: Randomly generated within variable bounds.
- Update mechanism: Variables are updated using Eq. (14):

14 $$\:{z}_{i+1,\:\:j,\:k}={z}_{i,\:\:j,\:k}+{\alpha\:\:}_{i,\:\:j,\:1}({z}_{i,\:\:j,\:B}-\left|{(z}_{i,\:\:j,\:k})\right|)-{\alpha\:\:}_{i,\:\:j,\:2}({z}_{i,\:\:j,\:W}-\:\left|{(z}_{i,\:\:j,\:k})\right|)\:$$

Where:

- $\:{z}_{i,\:\:j,\:B}$ and $\:{z}_{i,\:\:j,\:W}$ denote the indices of the population’s best and worst solutions, respectively.
- i, j, and k represent the indices of the candidate solution, variable, and iteration, respectively.
- $\:{z}_{i,\:\:j,\:k}$signifies the *j*^th^ variable of the *i*^*th*^ candidate solution in the *kth iteration*.
- $\:{\alpha\:\:}_{i,\:\:j,\:1}$ and $\:{\alpha\:\:}_{i,\:\:j,\:2}$are randomly generated numbers within [0, 1], acting as scaling factors for exploration.
- Absolute values of variables further promote exploration.

### Computational steps for the OPF problem

- Input: Population size (*P*), maximum iterations (*I*), control variables (*D*), and variable bounds (*X*_*min*_ and *X*_*max*_).
- Initialization: Set iteration count I = 0, randomly generate ‘*P’* initial solutions within bounds, and evaluate fitness using Eq. (13).
- Identify best and worst solutions: Determine $\:{z}_{i,\:\:j,\:B}$ and $\:{z}_{i,\:\:j,\:W}$based on fitness in the feasible search space.
- Generate new solutions: Apply Eq. (14) to create new candidate solutions.
- Acceptance: Accept new solutions if superior to previous ones, otherwise retain previous solutions.
- Iteration: If I < Imax, increment I and return to step 3. Otherwise, proceed to step 7.
- Output: Show the fitness value of the optimal solution.

## Numerical results and discussion

The efficacy of the proposed OPF algorithm was rigorously evaluated on three distinct test systems: the IEEE 30-bus, 57-bus, and 118-bus systems. In order to evaluate the functioning of the algorithm, 11 unique case studies were examined. The IEEE 30-bus system used a population size of 40, and maximum iteration limit of 100 is set. Subsequently, the IEEE 57-bus and IEEE 118-bus systems had these values increased to 50 and 150. The parameters were set this way to guarantee that the search space was correctly optimised while still being computationally effective. The simulations were carried out using a personal computer (PC) containing a 1.7 GHz Intel *Core* ***i***_***5***_ processor, 4GB RAM, and a 64-bit operating system, utilising the MATLAB computing environment.

### IEEE 30-bus test system

**Fig. 2:**
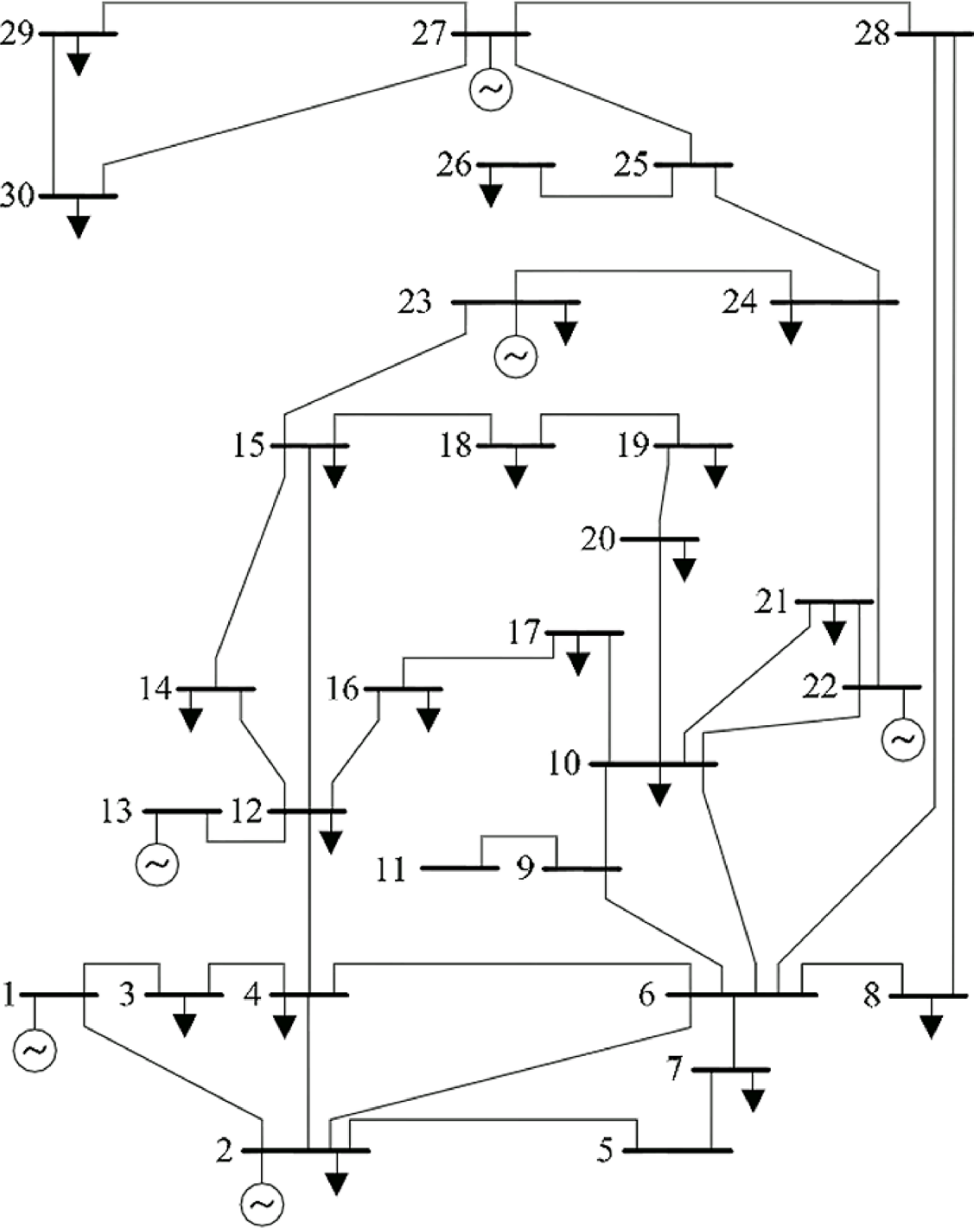
Single-line diagram of IEEE-30 Bus Test System.

The Jaya method is then tested with the same system for a total of 30 independent runs with varying objective functions of the OPF. The performance potential of the Jaya method at its optimum is then highlighted by displaying its best possible outcome amongst these runs. For a better comparison, PSO, DE, and TLBO algorithms were also tested with the same objective functions of the OPF with the same parameters, like population, number of generations and control variables. The control variables’ operational limits and specific system characteristics were taken from the standard benchmark data available in^30^. The IEEE-30 test bus system is shown in Fig. 2 (Table 2).

**Table 2:** Overview of OPF optimization cases with corresponding objectives.

Case no.	Objective(s)	Optimization type	Description
IEEE 30 Bus system			
Case 1	Fuel cost minimization	Single-objective optimization	Minimizes the total generation cost under standard OPF constraints
Case 2	Fuel cost + voltage profile improvement	Bi-objective optimization	Balances economic operation with voltage quality across the network
Case 3	Fuel cost + voltage stability enhancement	Bi-objective optimization	Simultaneously optimizes cost and enhances voltage stability
Case 4	Fuel cost + voltage stability enhancement during contingency	Bi-objective optimization	Simultaneously optimises cost, voltage stability enhancement during contingency
Case 5	Minimize the real power losses	Single-objective optimization	Minimizes the total power losses
Case 6	Emission minimization	Single-objective optimization	Reducing environmental impact from generation emissions
IEEE 57 Bus system			
Case 7	Fuel cost minimization	Single-objective optimization	Minimizes the total generation cost under standard OPF constraints
Case 8	Fuel cost + voltage profile improvement	Bi-objective optimization	Balances economic operation with voltage quality across the network
Case 9	Fuel cost + voltage stability enhancement	Single-objective optimization	optimizes voltage stability
Case 10	Minimize the real power losses	Single-objective optimization	Minimizes the total power losses
IEEE 118 Bus system			
Case 11	Fuel cost minimization	Single-objective optimization	Minimizes the total generation cost under standard OPF constraints

#### Case 1: Total fuel cost minimization

The objective is to reduce the price of generation/fuel cost. It can be described as^31^:

15 $$\:{\:\mathrm{F}}_{\mathrm{c}\mathrm{o}\mathrm{s}\mathrm{t}}\left(\mathrm{x},\mathrm{u}\right)={\sum\:}_{\mathrm{i}=1}^{\mathrm{N}\mathrm{G}\mathrm{N}\:\:}{\mathrm{A}}_{\mathrm{i}}+{\mathrm{B}}_{\mathrm{i}}{\mathrm{P}}_{{\mathrm{g}}_{\mathrm{i}}}+{\mathrm{C}}_{\mathrm{i}}{\mathrm{P}}_{{\mathrm{g}}_{\mathrm{i}}}^{2}\:\:(\mathrm{\$}/\mathrm{h})\:\:\:\:$$

**Fig. 3:**
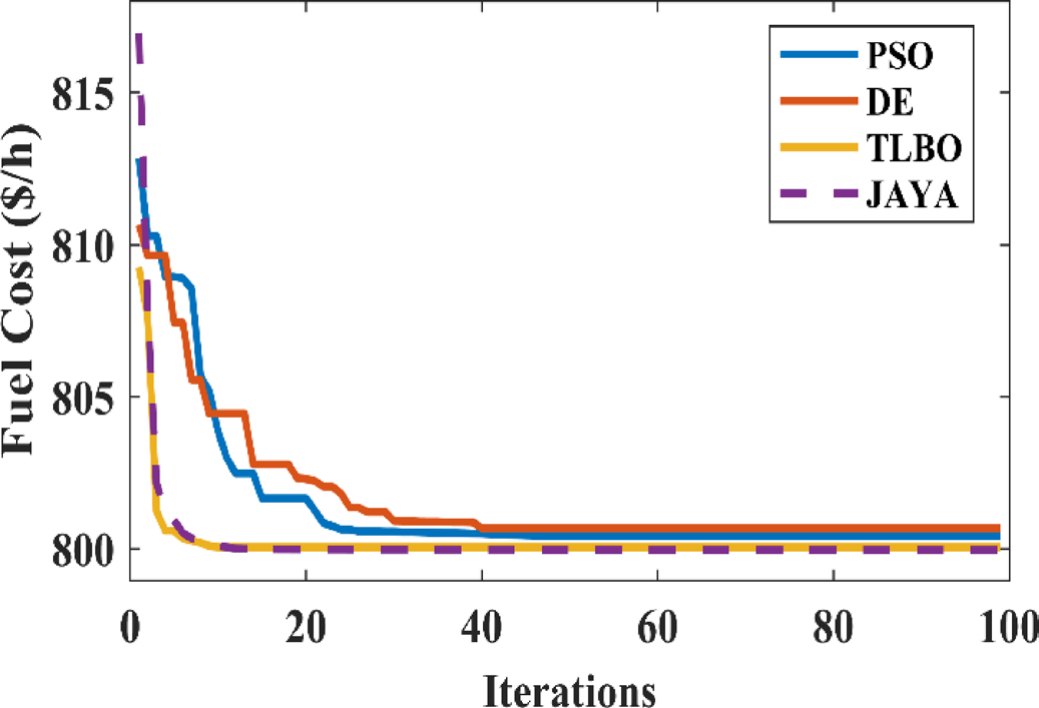
Convergence characteristics of Case 1.

**Fig. 4:**
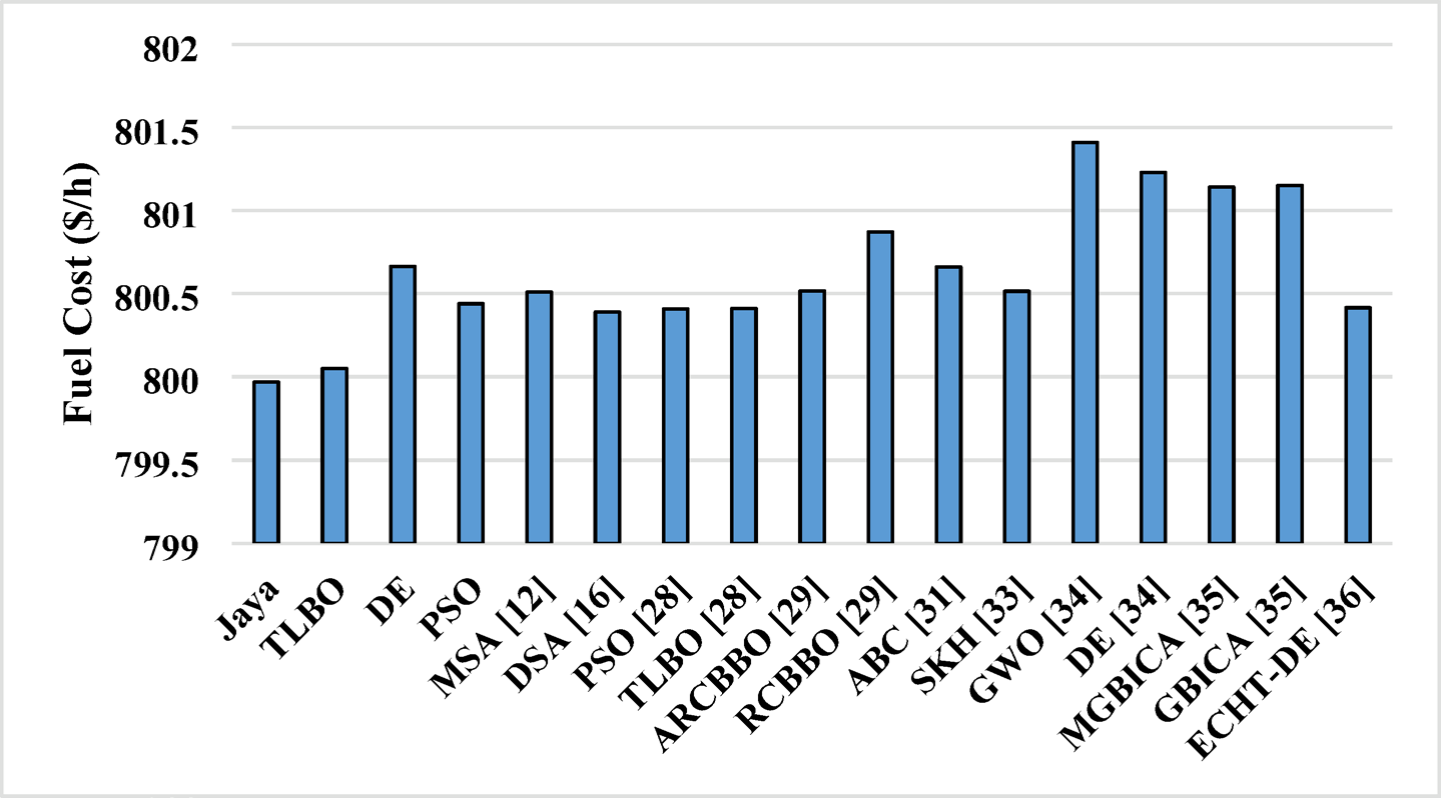
Comparative analysis of optimized OPF results with different algorithms.

In the direct competition for the minimum fuel cost, Jaya outperformed all others. Equipped with the fuel cost coefficients in^31^, Jaya thus obtained an outstanding result of 799.9676 $/h, while the others trailed behind in the competition: PSO (800.4391 $/h), DE (800.6636 $/h), and TLBO (800.0492 $/h). A full comparison of the performance is seen in Table 3 below, where the outstanding optimizing abilities of Jaya are seen in its performance compared to the existing algorithms. The convergence patterns of case 1 are seen in the picture below in Fig. 3. The results indicate that the Jaya algorithm provides competitive performance in minimizing fuel cost; however, it does not consistently outperform all benchmark methods across all evaluation metrics. The optimized fuel cost, compared with other algorithms, is presented in Fig. 4.

**Table 3:** Comparison of Case 1 numerical results.

Algorithm	FC	EC	PLOSS	VD	LI	CT
NR (Base Case)	902.004	0.222	5.842	1.160	0.1772	29.8312
**Jaya**	**799.9676**	**0.3358**	**8.9137**	**1.0988**	**0.1286**	**89.8123**
TLBO	800.0492	0.3357	8.9410	1.1481	0.1280	92.1267
DE	800.6636	0.3370	9.1490	0.8960	0.1302	94.6523
PSO	800.4391	0.3362	9.0613	0.9714	0.1307	99.7621
MSA^12^	800.5099	0.36645	9.0345	0.90357	0.13833	–
DSA^16^	800.3887	0.36672	8.9819	-	0.12624	–
PSO^28^	800.4075	0.366	9.006	0.9129	0.1276	–
TLBO^28^	800.4104	0.3663	9.008	0.9068	0.1269	–
ARCBBO^32^	800.5159	0.3663	9.0255	0.8867	0.1385	–
RCBBO^32^	800.8703	–	–	–	–	–
ABC^31^	800.66	0.36514	9.0328	0.9209	0.1381	–
SKH^33^	800.5141	0.3662	9.0282	–	0.1382	–
GWO^34^	801.41	–	9.3	–	–	–
DE^34^	801.23	–	9.22	–	–	–
MGBICA^35^	801.1409	0.3296	–	–	–	–
GBICA^35^	801.1513	0.3296	–	–	–	–
ECHT-DE^36^	800.4148	0.36593	8.9999	0.91231	0.13777	–

**CT= computation time in seconds.

#### Case 2: Bi-objective optimization – fuel cost with voltage profile improvement

The fuel cost function is commonly coupled with voltage profile improvement to construct a multi-objective formulation, represented in Eq. (16). The suggested objective function simultaneously addresses the following two aspects: minimization fuel expenses and the voltage deviations from 1 p.u. at all loads^31^.

16 $$\:{\:\mathrm{F}}_{\mathrm{V}\mathrm{D}}\left(\mathrm{x},\mathrm{u}\right)={\sum\:}_{\mathrm{i}=1}^{\mathrm{N}\mathrm{G}\mathrm{N}\:\:}{\mathrm{f}}_{\mathrm{i}}\left(.\right)\mathrm{\$}/\mathrm{h}+\:{{\upalpha\:}}_{\mathrm{V}\mathrm{D}}{\sum\:}_{\mathrm{i} \in \mathrm{N}\mathrm{L}}^{\:\:}\left|{\mathrm{V}}_{\mathrm{i}}-1\right|$$

Where $\:{{\upalpha\:}}_{\mathrm{V}\mathrm{D}}$ is a weight factor.

**Fig. 5:**
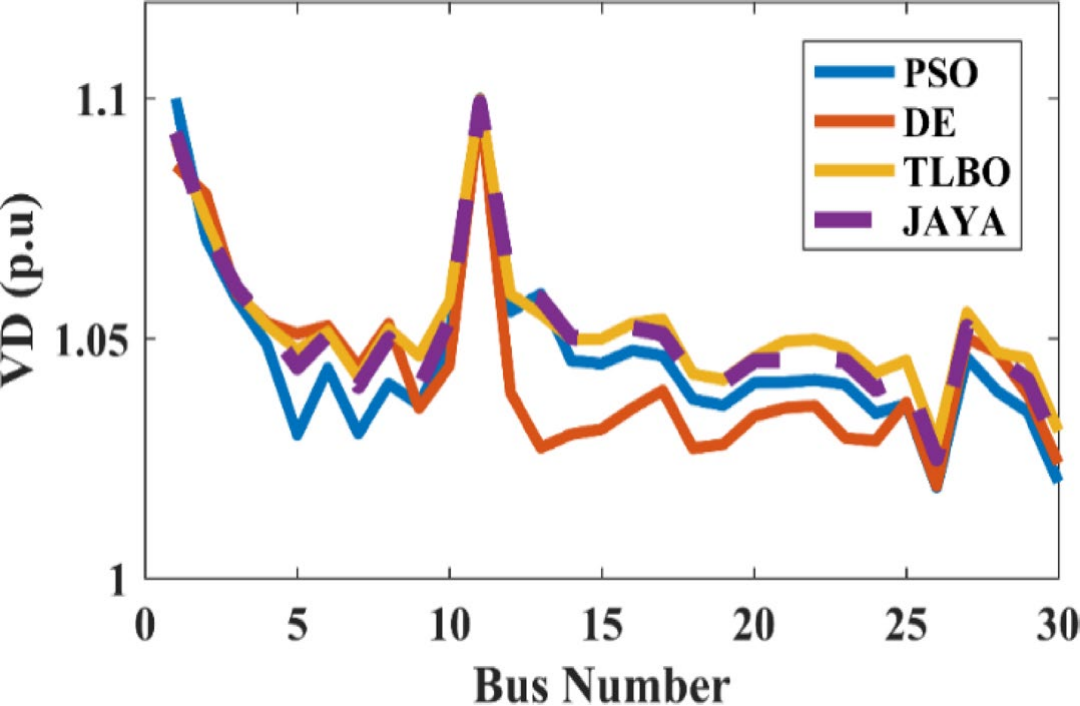
Convergence characteristics of Case 2.

The minimum voltage deviation is 0.0896 p.u., and a total fuel cost of 841.4409 $/h was found, respectively, while the voltage deviation obtained by PSO, DE, and TLBO algorithms is 0.0994, 0.0962, and 0.0906 p.u. respectively. The voltage profile is significantly improved in case 2. As earlier in case 1, the Total Voltage Deviation (TVD) was 1.0988 p.u, which is reduced to 0.0896 p.u. The optimized OPF results compared to other algorithms are provided in Table 4. In Fig. 5, the voltage profile is shown in case 1 and case 2.

**Table 4:** Comparison of Case 2 numerical results.

Algorithm	FC	EC	PLOSS	VD	LI	CT
NR (Base Case)	902.004	0.222	5.842	1.160	0.1772	29.8312
**Jaya**	**841.4409**	**0.4460**	**15.5359**	**0.0896**	**0.1402**	**92.1478**
TLBO	878.3593	0.3063	8.7408	0.0906	0.1411	95.0917
DE	804.5136	0.3331	10.0762	0.0962	0.1408	96.1176
PSO	853.1839	0.3275	13.4192	0.0994	0.1409	98.2271
MSA^12^	803.3125	0.3634	9.7206	0.1084	0.1478	–
MPSO^12^	803.9787	0.3636	9.9242	0.1202	0.149	–
PSO^28^	803.4736	0.3639	9.821	0.0978	0.1371	–
TLBO^28^	803.5675	0.3636	9.813	0.0939	0.1369	–
ECHT-DE^36^	803.7198	0.3638	9.8414	0.09454	0.14888	–
SF-DE^36^	803.4241	0.3642	9.7807	0.09772	0.14893	–
SP-DE^36^	803.4196	0.3632	9.7573	0.09776	0.14893	–

The weighted sum method (WSM), a classical and widely used scalarization technique in multi-objective optimization, is employed in this study to aggregate multiple objectives. While WSM is appropriate for problems with moderately aligned objectives, we acknowledge its limitations when objectives are strongly conflicting.

#### Case 3: Bi-objective optimization – fuel cost with voltage stability enhancement

The growing requirement for electricity exerts pressure on the power system because the generating capacity is unable to match it. The result of the growing gap between the two is a concerning “downward spiral situation,” where the lack of power causes low voltage conditions at the load buses, thereby further aggravating the voltage stability condition in the system. A critical requirement in a healthy power system is the regulation of the bus voltage within acceptable margins in the normal functioning condition as well as during the sudden change in the loading condition. All load buses employ the “L-index,” which ranges from 0 to 1, to monitor the system’s voltage stability conditions. Buses having greater L-index values are more prone to voltage instability. A significant improvement in the voltage stability conditions in the system can be achieved by lowering the “L-index,” a crucial metric for assessing the voltage conditions of the relevant buses^31^. The fuel cost function is integrated with L-index to formulate a combined objective function, as expressed in Eq. (17).

17 $$\:{{\mathrm{F}}_{\mathrm{L}\mathrm{I}}\left(\mathrm{x},\mathrm{u}\right)\:={\sum\:}_{\mathrm{i}=1}^{\mathrm{N}\mathrm{G}\mathrm{N}}{\mathrm{f}}_{\mathrm{i}}\left(.\right)\mathrm{\$}/\mathrm{h}+{{\upalpha\:}}_{\mathrm{L}}\mathrm{L}}_{\mathrm{m}\mathrm{a}\mathrm{x}}\:\:\:\:\:\:\:$$

The Jaya algorithm resulted in a minimum L-index of 0.1283 and a corresponding total fuel cost of 841.4409 $/h. The Jaya approach presents better results than TLBO, DE, PSO and other reported methods. The optimized OPF results compared to other algorithms are provided in Table 5. The weighted sum method is used in this study due to its simplicity; we recognize that it may not fully capture the Pareto structure of the OPF problem when objectives are strongly conflicting.

**Table 5:** Comparison of Case 3 numerical results.

Algorithm	FC	EC	PLOSS	VD	LI	CT
NR (Base Case)	902.004	0.222	5.842	1.160	0.1772	29.8312
**Jaya**	**810.2319**	**0.3408**	**11.9651**	**0.6223**	**0.1283**	**96.2789**
TLBO	807.1304	0.3390	11.0079	0.5574	0.1305	100.2611
DE	809.8085	0.3326	11.4177	0.3327	0.1332	99.8901
PSO	808.0029	0.3395	11.2978	0.3765	0.1298	102.1098
MSA^12^	801.2248	0.3611	8.9761	0.9265	0.1371	–
IMFO^28^	800.4762	0.3662	9.016	0.9086	0.1255	–
ECHT-DE^36^	800.4321	0.36585	9.0043	0.91244	0.13739	–
SF-DE^36^	800.4203	0.36592	8.9985	0.93846	0.13745	–
SP-DE^36^	800.4365	0.36517	8.9838	0.93706	0.13748	–

#### Case 4: Bi-objective optimization – fuel cost with improvement of voltage stability during contingency

Regular power system operations experience unplanned events like transmission line failures and generator blackouts, which are termed contingencies. The configuration of a system can change due to contingencies that may impact voltage stability levels^31^. The objective of Case 4 addresses voltage stability enhancement alongside minimization of fuel cost under scenarios involving single-line outages at buses 2–6.

This is to retain operability despite contingencies or disturbances. The proposed technique does an excellent job in handling contingencies, along with optimizing fuel cost and voltage stability. Case 4 is devoted to studies of (n-1) contingencies, taking into consideration single-line outage contingencies for a robust operational system. Under this approach, a minimum value of the L-index was achieved at 0.1363 with a fuel cost of $818.5353/h, outperforming algorithms such as TLBO, DE, and PSO, where the L-indices were 0.1469, 0.1439, and 0.1485, respectively. Table 6 presents comparisons with some other evolutionary computation-based algorithms.

**Table 6:** Comparison of Case 4 numerical results.

Algorithm	FC	EC	PLOSS	VD	LI	CT
NR (Base Case)	902.004	0.222	5.842	1.160	0.1772	29.8312
**Jaya**	**818.5353**	**0.3375**	**14.145**	**0.7439**	**0.1363**	**101.9081**
TLBO	825.3808	0.2800	9.3870	0.6317	0.1469	108.2716
DE	810.3012	0.3324	11.3868	0.5754	0.1439	107.1101
PSO	827.3375	0.2792	9.2645	0.5925	0.1485	109.2651
MSA^12^	804.4838	0.3613	9.9486	0.9177	0.1392	–

#### Case 5: Minimize the real power losses

In this section, real power loss minimization was selected as the main objective function. The mathematically $\:{F}_{\mathrm{l}\mathrm{o}\mathrm{s}\mathrm{s}}$ can be represented as follows:

18 $$\:{\mathrm{F}}_{\mathrm{L}\mathrm{O}\mathrm{S}\mathrm{S}}\left(\mathrm{x},\mathrm{u}\right){\sum\:}_{\mathrm{k}=1}^{\mathrm{N}\mathrm{T}\:}{\mathrm{G}}_{\mathrm{k}}\left|{\mathrm{v}}_{\mathrm{i}}^{2}+{\mathrm{v}}_{\mathrm{j}}^{2}+2\left|{\mathrm{V}}_{\mathrm{i}}\right|\left|{\mathrm{V}}_{\mathrm{j}}\right|\mathrm{cos}({{\updelta\:}}_{\mathrm{i}}-{{\updelta\:}}_{\mathrm{j}})\:\right|$$

Where $\:{\mathrm{G}}_{\mathrm{k}}\:$is the conductance of the kth line connected between the ith and jth buses: NT is the number of transmission lines: $\:{\mathrm{V}}_{\mathrm{i}\:}$is the voltage magnitude at bus i: $\:{\mathrm{V}}_{\mathrm{j}}$is the magnitude at bus j: $\:{{\updelta\:}}_{\mathrm{i}}$ is the voltage angles at bus i: $\:{{\updelta\:}}_{\mathrm{j}}$ is the voltage angle at bus j.

**Fig. 6:**
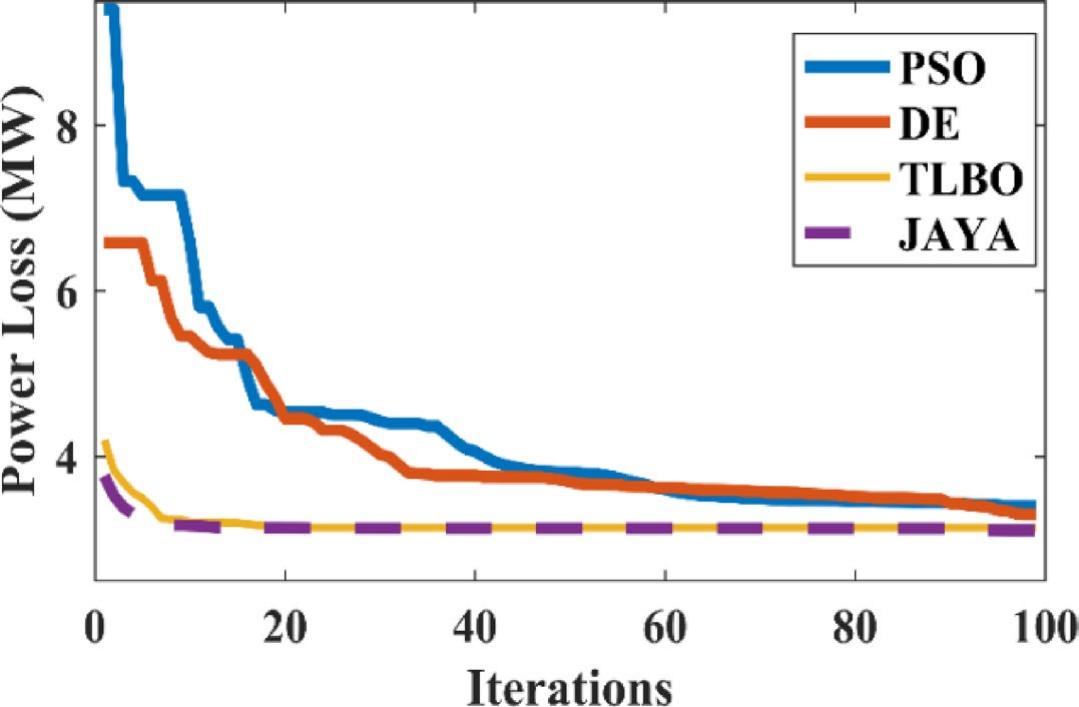
Convergence characteristics of Case 5.

The$\:{\:\mathrm{F}}_{\mathrm{L}\mathrm{o}\mathrm{s}\mathrm{s}\:}$is selected for the real power loss minimization as described in Eq. (18). Here, the nature of all generator cost characteristics is quadratic. The numerical results found after optimization were compared with PSO, DE, TLBO, and other reported results, as given in Table 7. In the present case, the Jaya algorithm gives 3.1086 MW after optimization which is slightly higher than the result obtained by MSA, MPSO, IMFO, PSO, and TLBO methods. The convergence characteristic obtained by the Jaya algorithm for real power loss minimization is represented in Fig. 6. In this Case, the Jaya optimizer yields a result that is slightly inferior to other competing algorithms. This behavior is attributed to (i) the conflicting nature of OPF objectives under the weighted-sum formulation, (ii) the sensitivity of local search dynamics to penalty/constraint interactions, and (iii) the inherent trade-offs between exploration and exploitation. Despite this, the Jaya algorithm maintains overall competitive performance across all test cases and demonstrates stable convergence with lower computational complexity.

**Table 7:** Comparison of Case 5 numerical results.

Algorithm	FC	EC	PLOSS	VD	LI	CT
R (Base Case)	902.004	0.222	5.842	1.160	0.1772	29.8312
**Jaya**	**967.6830**	**0.2066**	**3.1086**	**1.0361**	**0.1302**	**94.1191**
TLBO	967.7550	0.2066	3.1388	1.0011	0.1294	98.1141
DE	968.1496	0.2066	3.3041	0.4125	0.1391	96.1109
PSO	968.5248	0.2066	3.4612	0.7107	0.1418	99.8601
MSA^12^	967.6636	0.20727	3.1005	0.8886	0.1385	–
MPSO^12^	967.6523	0.20727	3.1031	0.9063	0.1381	–
TLBO^28^	967.5841	0.2073	3.088	0.9029	0.1274	–
SKH^33^	967.6594	0.2072	3.0987	NA	0.1385	–
ECHT-DE^36^	967.6001	0.20726	3.0850	0.90937	0.13836	–
SF-DE^36^	967.6198	0.20726	3.0845	0.90648	0.13818	–
SP-DE^36^	967.5962	0.20726	3.0844	0.90359	0.13832	–

#### Case 6: Total emission cost minimization

The power plant’s emissions of the key pollutants SOx and NOx are monitored closely. These gases are produced by all generating units and their output is estimated using a combination of exponential and quadratic functions based on each unit’s active power. The total emission cost is then determined based on these calculations^31^.

19 $$\:{\mathrm{F}}_{\mathrm{E}\mathrm{C}\mathrm{O}\mathrm{S}\mathrm{T}}\left(\mathrm{x},\mathrm{u}\right)={\sum\:}_{\mathrm{i}=1}^{\mathrm{N}\mathrm{G}\:\:}{{\upalpha\:}}_{\mathrm{i}}+\:{{\upbeta\:}}_{\mathrm{i}}{\mathrm{P}}_{{\mathrm{G}}_{\mathrm{i}}}+\:{{\upgamma\:}}_{\mathrm{i}}{\mathrm{P}}_{{\mathrm{G}}_{\mathrm{i}}}^{2}+\:\:{{\upxi\:}}_{\mathrm{i}}\mathrm{exp}\left({{\uplambda\:}}_{\mathrm{i}}{\mathrm{P}}_{{\mathrm{G}}_{\mathrm{i}}}\right)\left(ton/h \right)\:\:\:$$

**Fig. 7:**
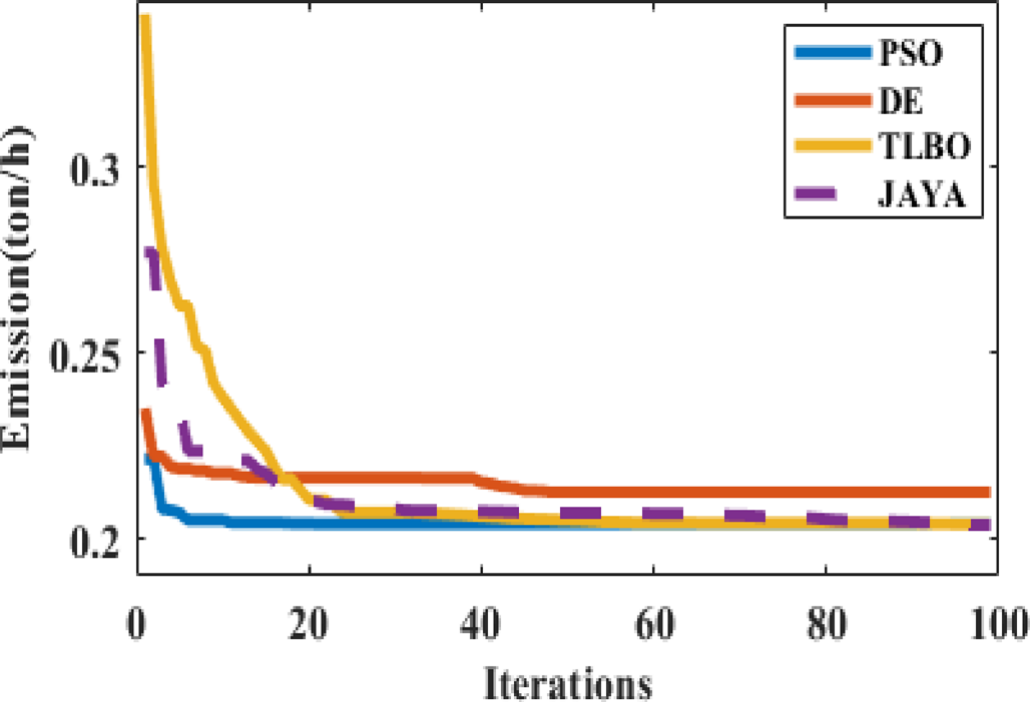
Convergence characteristics of Case 6.

The$\:{\:{\upalpha\:}}_{\mathrm{i}}$,$\:{{\upbeta\:}}_{\mathrm{i}}$,$\:{{\upgamma\:}}_{\mathrm{i}}$,$\:{{\upxi\:}}_{\mathrm{i}}$ and $\:{{\uplambda\:}}_{\mathrm{i}}$ are the emission coefficients. The $\:{\mathrm{F}}_{\mathrm{E}\mathrm{C}\mathrm{O}\mathrm{S}\mathrm{T}}\left(\mathrm{x},\mathrm{u}\right)$is selected for total emission cost optimisation as described in (19). Here, the nature of the generating cost characteristics is quadratic. As earlier in case 1, the emission cost was 0.3358 ton/h, which is reduced to 0.2037 ton/h (39.33%) in case 6. The simulation results of PSO, DE, TLBO, and other methods mentioned in the literature are compared with the Jaya algorithm shown in Table 8. It is seen that the results from the suggested method are excellent in comparison to other methods. The convergence characteristic is shown in Fig. 7. Appendix: Table 15 lists the Jaya algorithm control variables for Cases 1–6.

**Table 8:** Comparison of case 6 numerical results.

Algorithm	FC	EC	PLOSS	VD	LI	CT
NR (Base Case)	902.004	0.222	5.842	1.160	0.1772	29.8312
**Jaya**	**942.3443**	**0.2037**	**3.2162**	**1.1261**	**0.1286**	**97.1118**
TLBO	944.3374	0.2039	3.7807	0.6757	0.1402	96.7198
DE	915.2185	0.2126	4.2325	0.5493	0.1467	98.0191
PSO	944.1722	0.2040	3.9623	0.8210	0.1328	100.0914
MSA^12^	944.3904	0.20482	3.2361	0.88059	0.1387	–
DSA^16^	944.4086	0.2058255	3.24373	–	0.12734	–
TLBO^28^	944.3278	0.2048	3.221	0.8988	0.1271	–
ABC^31^	944.4391	0.204826	3.247	0.8463	0.1402	–
SKH^33^	944.3802	0.2048	3.2326	–	0.1384	–
MGBICA^35^	942.8401	0.2048	–	–	–	–
GBICA^35^	944.6516	0.2049	–	–	–	–
ECHT-DE^36^	944.3782	0.20482	3.2179	0.89240	0.13844	–
SF-DE^36^	944.3242	0.20482	3.2179	0.89617	0.13844	–
SP-DE^36^	944.3323	0.20482	3.2178	0.90215	0.13830	–
MAHA^37^	943.9774	0.2047	3.2035	0.1273	0.8765	–
MGTO^38^	943.2500	0.2047	2.9218	2.9783	0.1060	–
MARO^39^	943.808	0.20479	3.236	0.7668	0.1296	–

### IEEE 57-bus system

**Fig. 8:**
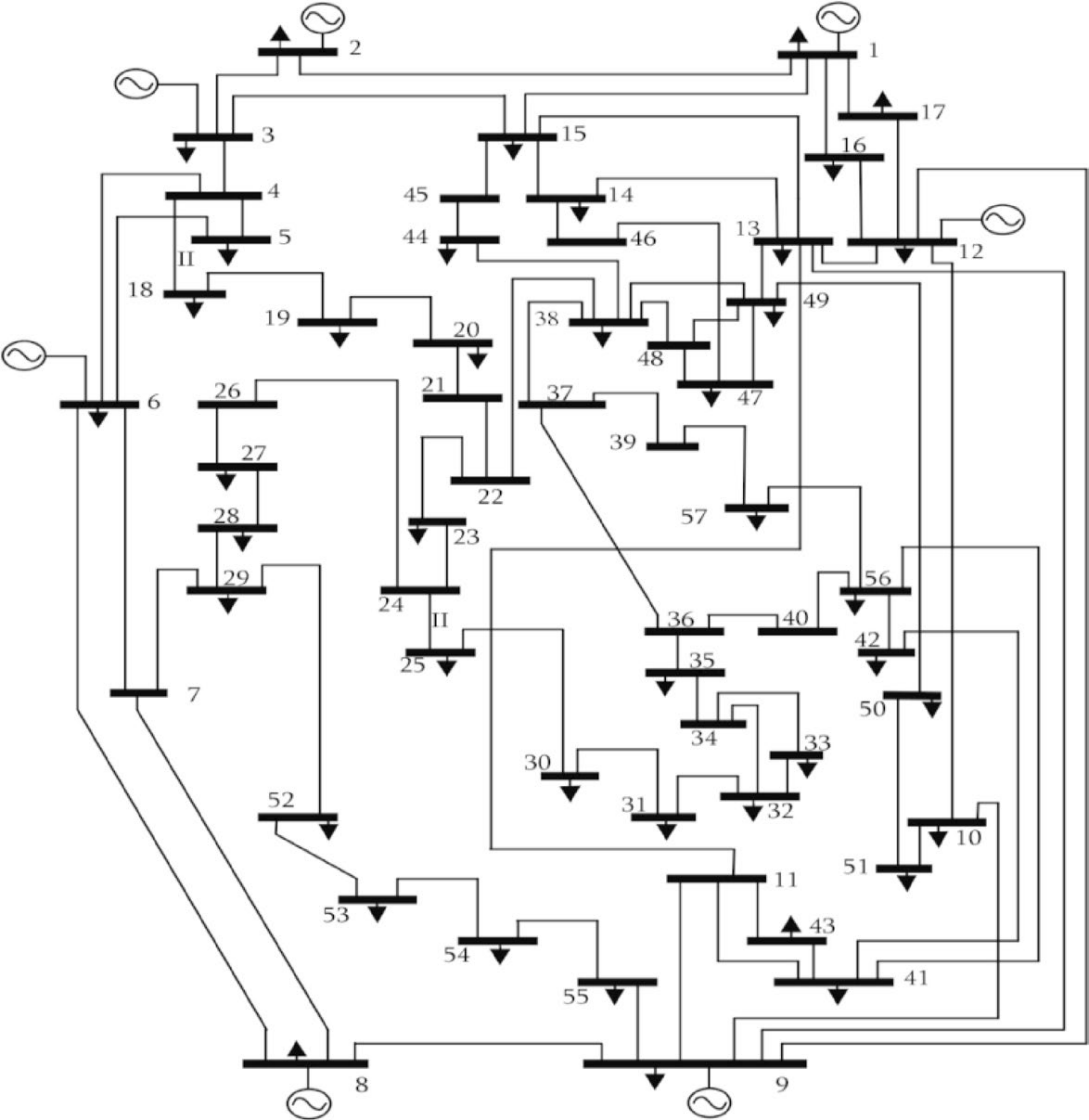
Single-line diagram of the IEEE-57 Bus Test System.

The effectiveness of the Jaya algorithm was evaluated on the well-known IEEE 57 bus system, a benchmark for power system optimization. Detailed system parameters and operating limits of the control variables were adopted from standard benchmark data reported in^40^. The IEEE-57 test bus system is shown in Fig. 8.

#### Case 7: Total fuel cost minimization

**Fig. 9:**
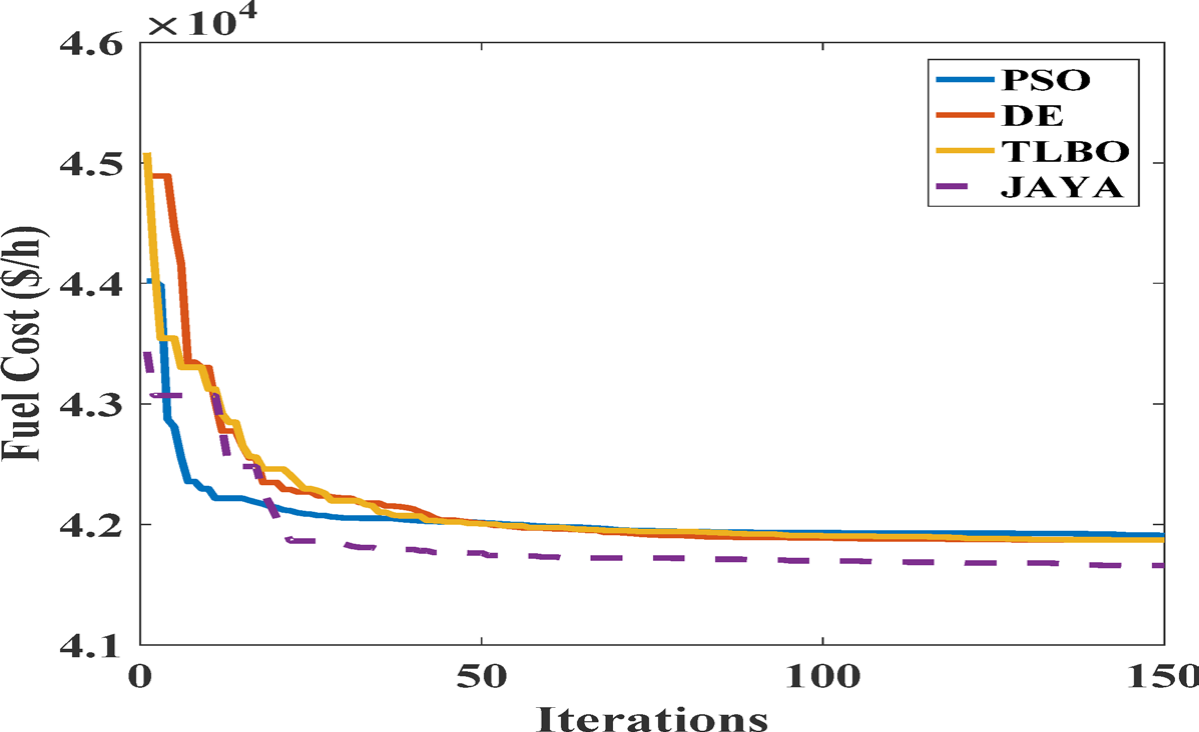
Convergence Characteristics of Case 7.

**Fig. 10:**
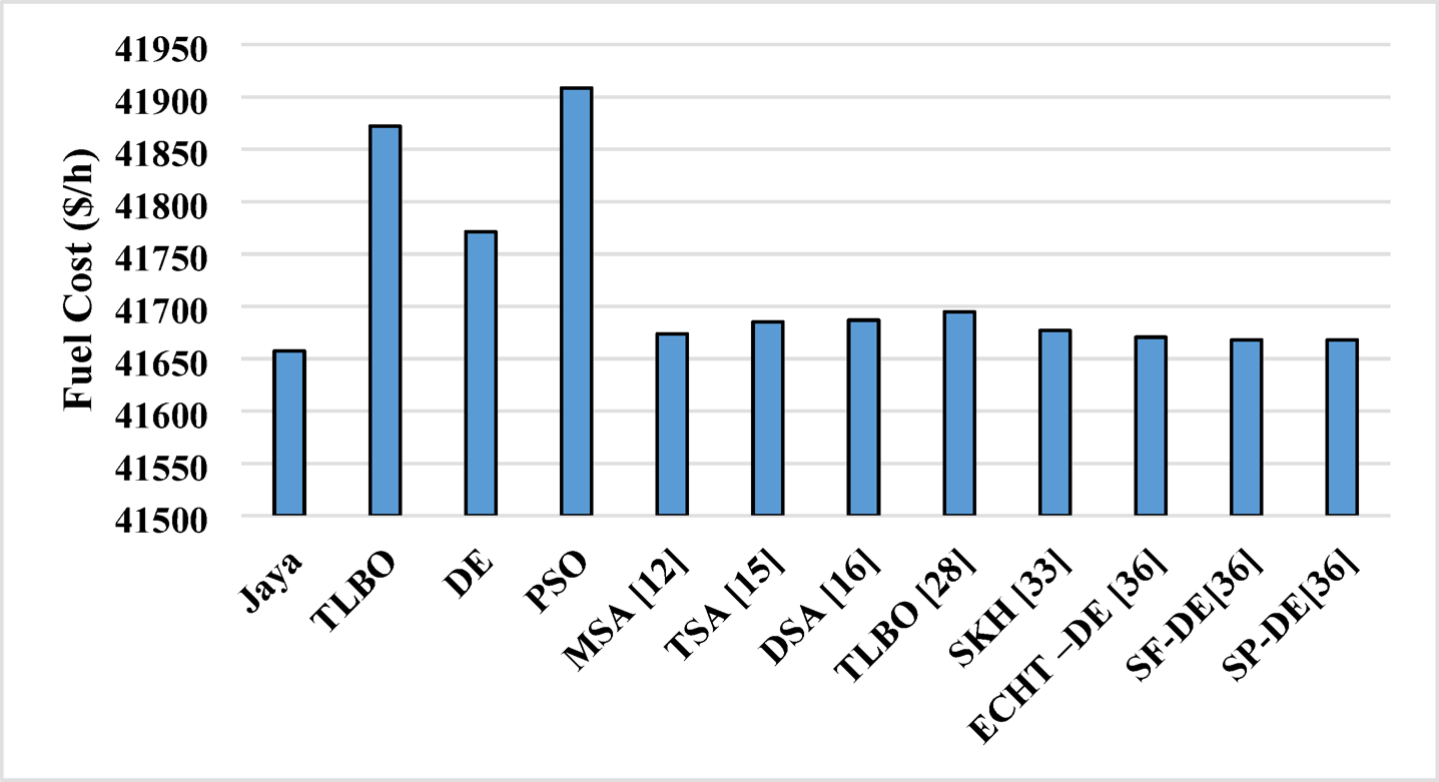
A comparative representation of the optimized fuel cost with different algorithms.

Minimizing total fuel cost was the primary objective, as in case 1, and the objective function was defined in Eq. (15). All generating cost characteristics were quadratic. The Jaya algorithm achieved the optimal fuel cost of $41657.3521/h, outperforming other reported results. Notably, TLBO, DE, and PSO algorithms obtained costs of $41,872.0668/h, $41,771.1088/h, and $41,909.5363/h, respectively. Table 9 clearly demonstrates the dominance of the Jaya algorithm’s results compared to other methods. Figure 9 illustrates the fuel cost characteristic for case 7. A comparative representation of the optimized fuel cost with different algorithms is illustrated in Fig. 10.

**Table 9:** Comparison of Case 7 numerical results.

Algorithm	Fuel Cost	Ploss	VD	L-index	CT
NR (Base Case)	51,395.57064	28.36589	1.25517	0.3099	40.7423
**Jaya**	**41657.3521**	14.7339	1.7122	0.2327	**130.2561**
TLBO	41872.0668	16.4837	1.6713	0.2411	132.9081
DE	41771.1088	17.3664	1.5465	0.2310	132.9871
PSO	41908.5363	16.2962	1.0263	0.2527	134.8891
MSA^12^	41673.7231	15.0526	1.5508	0.28392	–
TSA^15^	41,685.07	16.973	2.92	–	–
DSA^16^	41686.82	–	0.24353	1.0833	–
TLBO^28^	41694.7778	15.7426	0.712	0.2409	–
SKH^33^	41676.9152	15.0795	–	0.2807	–
ECHT –DE^36^	41670.562	14.9479	0.50319	0.28886	–
SF-DE^36^	41667.85	14.8864	1.64209	0.27971	–
SP-DE^36^	41667.82	14.909	1.54367	0.28123	–

#### Case 8: Bi-objective optimization – fuel cost with voltage profile improvement

**Fig. 11:**
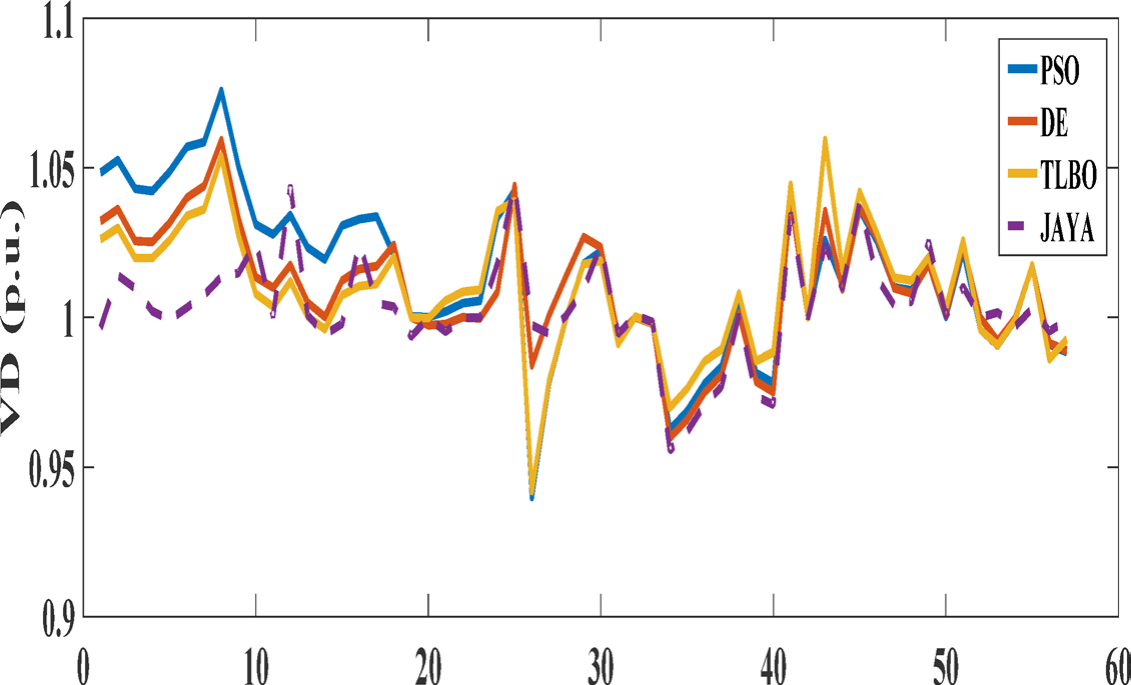
Convergence characteristics of Case 8.

For a stable power system, the bus voltages should satisfy the standard of 1 p.u. In this condition, the objective function sought to concurrently reduce the total cost and enhance the voltage profile, as explained by Eq. (16). In this study, the Jaya method is effective in obtaining a minimal voltage variation of 0.5889p.u. with a fuel cost of $41755.6721/h. The optimized values of the results are shown in Table 10. Case 8 led to a considerable improvement in the voltage profiles. From a total voltage deviation of 1.6953 p.u. in case 7, a highly decreased TVD of 0.5725 p.u. has been achieved in case 8. The results of voltage deviation for the proposed algorithm are compared with the outcomes of PSO, DE, TLBO, and other existing algorithms in Table 10. The convergence curve for this case is illustrated in Fig. 11.

**Table 10:** Comparison of Case 8 numerical results.

Algorithm	Fuel Cost	Ploss	VD	L-index	CT
**NR (base case)**	51,395.57064	28.36589	1.25517	0.3099	40.7423
**Jaya**	**41755.6721**	**16.6558**	**0.5889**	**0.2348**	**131.8891**
TLBO	41710.3834	15.9215	0.8007	0.2443	133.4912
DE	4169.1102	15.4214	0.7644	0.2414	133.8912
PSO	41688.4417	15.4719	0.9882	0.2438	134.0211
FPA^12^	41726.3758	16.027	0.69723	0.29488	–
TSA^15^	54,045.17	28.513	0.72	–	–
DSA^16^	41699.4	–	0.762	0.2471	–
IMFO^28^	41692.7178	15.7552	0.7182	0.24259	–
ECHT-DE^36^	41694.82	15.5806	0.81659	0.29198	–
SF-DE^36^	41697.52	15.5616	0.77572	0.29262	–
SP-DE^36^	41697.5	15.5897	0.77253	0.29228	–

#### Case 9: Voltage stability enhancement

With both aims of fuel cost and voltage stability improvement, the objective function was expressed by Eq. (16). The Jaya method was successful and achieved a minimum value of the L-index of 0.2200, signifying a substantial improvement in voltage stability. A comparison of numerical results of both fuel cost and minimum value of L-index using proposed Jaya, TLBO, DE, PSO, and other recently developed meta-heuristic algorithms is provided in Table 11. The results truly revealed that the Jaya method performs better than other methods existing in the literature.

**Table 11:** Comparison of Case 9 results.

Algorithm	Fuel Cost	Ploss	VD	L-index	CT
NR (Base Case)	51,395.57064	28.36589	1.25517	0.3099	40.7423
**Jaya**	**41665.3565**	**14.801**	**1.8417**	**0.2200**	**132.9142**
TLBO	41692.9720	15.561	1.7835	0.2191	132.9912
DE	41814.9630	17.3643	1.5748	0.2459	133.9084
PSO	41670.47269	15.0175	1.7637	0.2200	134.9071
MSA^12^	41675.9948	15.0026	1.7236	0.27481	–
MPSO^12^	41694.1407	15.4554	1.5084	0.27918	–
MDE^12^	41689.5878	15.7092	1.5447	0.27677	–
MFO^12^	41680.1937	15.1026	1.7245	0.27467	–
FPA^12^	41684.1859	15.2193	1.7478	0.27429	–
DSA^16^	41761.22	–	1.0573	0.2383	–
IMFO^28^	41673.6204	15.299	1.17625	0.23525	–
MFO^28^	41688.6522	15.475	1.3988	0.2395	–
GA^28^	41670.0872	15.165	1.2473	0.2413	–
PSO^28^	41670.1755	15.205	1.4323	0.242	–
TLBO^28^	41685.353	15.478	1.3453	0.24787	–
SKH^33^	43937.1058	13.3154	–	0.2721	–
ECHT-DE^36^	41671.09	15.0275	1.56188	0.28152	–
SF-DE^36^	41667.53	14.8963	1.61174	0.28022	–
SP-DE^36^	41668.45	15.012	1.60803	0.28092	–

#### Case 10: Minimize the real power losses

**Fig. 12:**
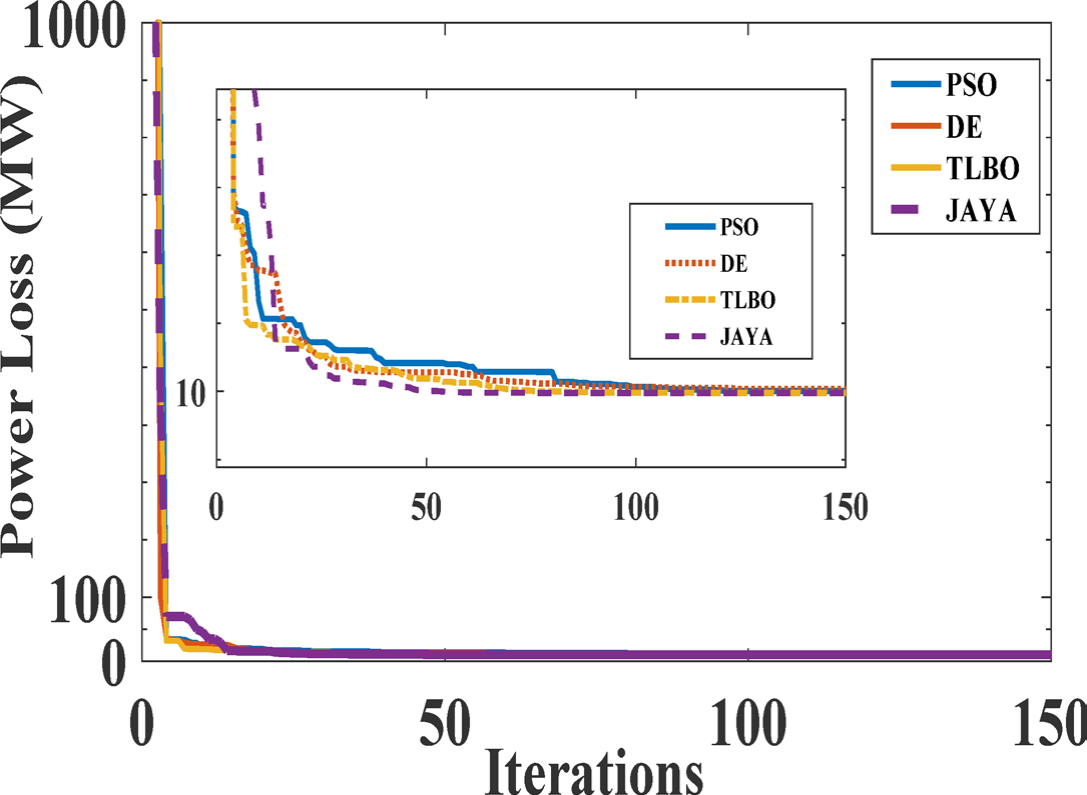
Convergence characteristics of Case 10.

Minimizing real power loss was the objective, as in case 5. The Jaya algorithm achieved optimal values of 9.7924 MW for real power loss and $4441.4980/h for fuel cost, outperforming TLBO, DE, and PSO algorithms with losses of 9.770, 10.30, and 10.00 MW, respectively. Figure 8 shows the convergence characteristic for real power loss minimization. Table 12 compares Jaya’s OPF results with other evolutionary computation algorithms. Appendix: Table 16 lists the Jaya algorithm control variables for Cases 7–10 (Fig. 12).

**Table 12:** Comparison of Case 10 numerical results.

Algorithm	Fuel cost	Ploss	VD	L-index	CT
NR (Base Case)	51,395.57064	28.36589	1.25517	0.3099	40.7423
**Jaya**	**4441.4980**	**9.7924**	**1.7192**	**0.2348**	**134.0962**
TLBO	44,438	9.7700	1.7464	0.2353	136.1989
DE	44,747	10.3000	1.2495	0.2435	135.8910
PSO	44,418	10.0000	1.5278	0.2434	135.0914
TSA^15^	44,519.07	12.473	2.69	–	–
SKH^33^	45044.2407	10.6877	–	0.2857	–

### IEEE 118-bus test system

**Fig. 13:**
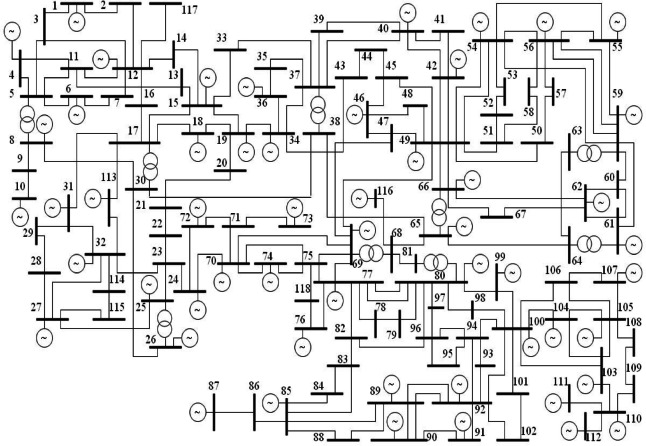
Single-line diagram of IEEE-118 Bus Test System.

To evaluate the scalability and effectiveness of Jaya for large-scale power systems, Jaya was applied to the IEEE 118-bus system. This benchmark system consists of 54 generating units, two shunt reactors, twelve capacitors, 186 transmission lines, and nine tap-changing transformers. Detailed system parameters and operating limits of the control variables were adopted from standard benchmark data reported in^41^. Jaya was executed for 30 independent trials to ensure statistical reliability, and the best-performing solutions among these runs are reported in this section. The IEEE-118 test bus system is shown in Fig. 13.

#### Case 11: Fuel cost minimization

**Fig. 14:**
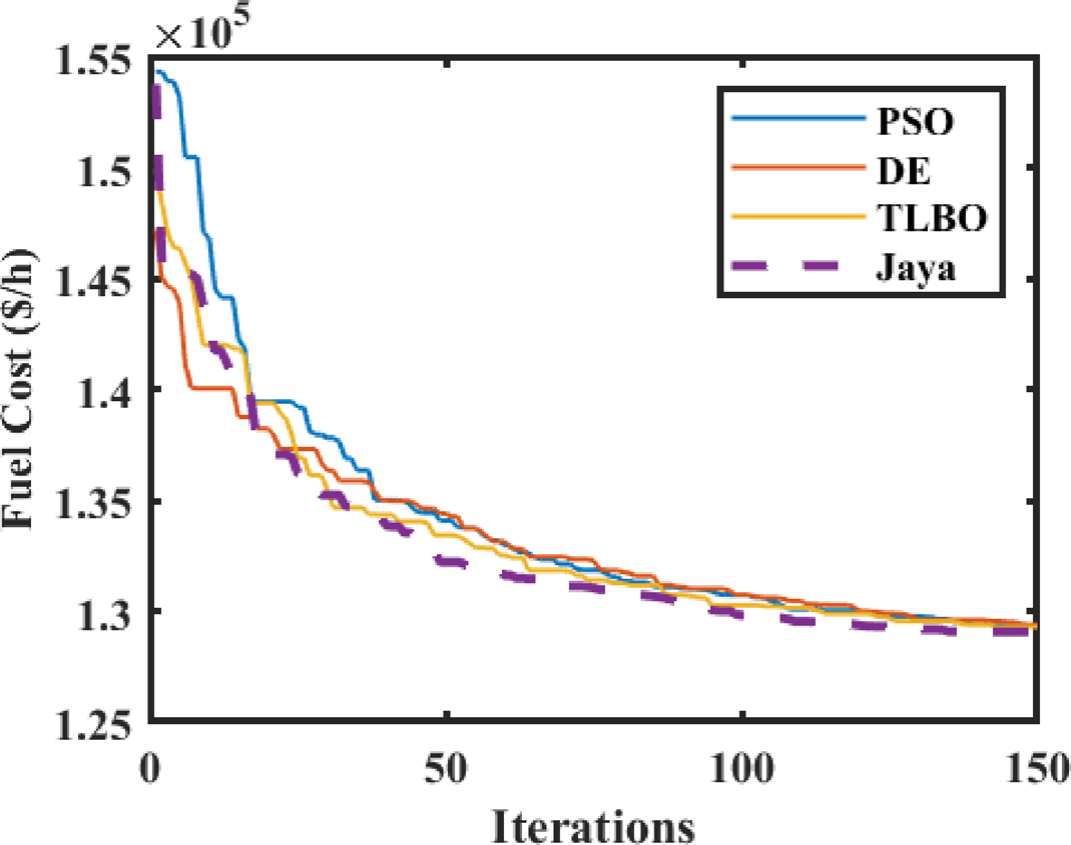
Convergence Characteristics of Case 11.

**Fig. 15:**
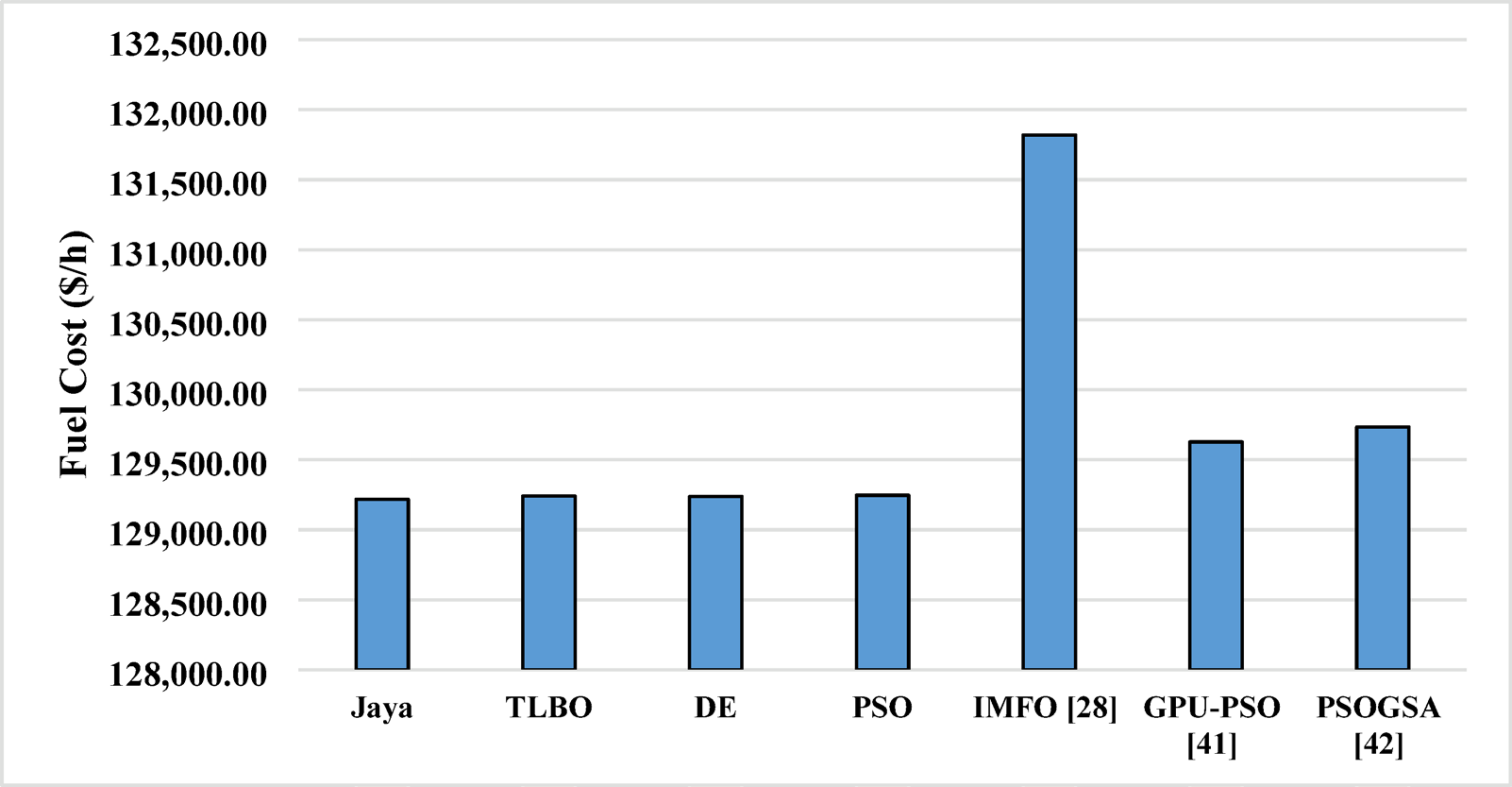
A comparative representation of the optimized fuel cost with different algorithms.

In Case 11, minimization of generation fuel cost was considered as the sole objective. The Jaya algorithm achieved the lowest operating cost of **129**,**216.3118 $/h**, whereas TLBO, DE and PSO resulted in minimum costs of **129**,**240.3789 $/h**,**129**,**236.9901 $/h** and **129**,**246.1098 $/h**, respectively. A comparative analysis of the OPF results obtained using the Jaya algorithm and other recently reported approaches is provided in Table 13. The comparison clearly indicates that Jaya provides the most economical solution among all evaluated techniques, confirming its better optimization capability. The convergence characteristics of fuel cost for case 11 are shown in Fig. 14. Figure 15 shows a comparison of optimized fuel cost with various algorithms.

**Table 13:** Comparison of Case 11 numerical results.

Algorithm	Fuel cost	CT
NR (Base Case)	131,220.0208	90.1287
**Jaya**	**129**,**216.3118**	**162.8871**
TLBO	129,240.3789	166.9812
DE	129,236.9901	168.1192
PSO	129,246.1098	163.1231
IMFO^28^	131820.0	–
GPU-PSO^41^	129,627.03	–
PSOGSA^42^	129,733.58	–

## Statistical analysis

Since stochastic methods are based on random behaviour in nature, running these methods several times independently for the same problem is necessary to achieve better results because the results can vary from one run to another. To perform a fair and unbiased comparison of the proposed algorithm with other competing algorithms, a one-way ANOVA test was used as a statistical tool to check the performance of the proposed optimizer algorithm.

**Fig. 16:**
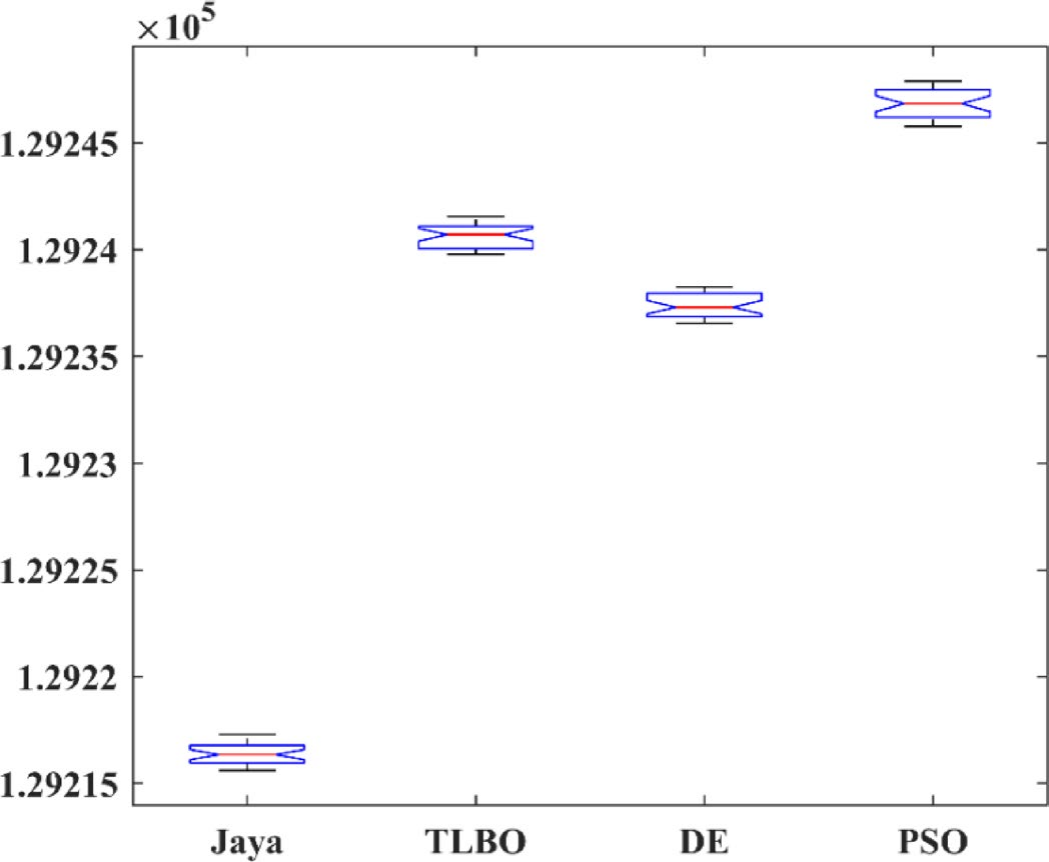
One-way ANOVA comparison of OPF algorithms for IEEE 118 Bus System.

In this study, the value of the significance level is set to 0.05. The result, where the p-value is less than 0.05, shows that the difference observed in the algorithms is statistically significant and not due to any variation. Therefore, this test is important in proving the effectiveness and robustness of the suggested approach. Table 14 shows the result obtained from the one-way ANOVA test, which was conducted on all optimizers on the IEEE 118 bus system. The result shows that the obtained p-value is less than 0.05, indicating clearly that there is a difference between the algorithms. Moreover, from the better result, it is deduced that the Jaya algorithm is better than the others with a high level of statistical confidence. Figure 16 shows the one-way ANOVA analysis of the OPF algorithms on the IEEE 118 bus system.

**Table 14:** Statistical results of the one-way ANOVA test for the IEEE 118 bus system.

Source	SS	df	MS	F	Prob > F
Groups	15710.9	3	5236.96	15407.59	1.12256e−150
Error	39.4	116	0.34		
Total	15750.3	119			

## Discussion

The experimental results obtained for the OPF optimization problem show that the optimization approach can achieve the minimum value for the objective functions while maintaining stable convergence characteristics. The convergence characteristics show that the algorithm performs an initial wide range of exploration followed by gradual exploitation in the search space. This is important in solving OPF optimization problems due to the presence of several local optima in the solution space, due to generator limits, power balance conditions, and transmission limits. As can be seen from the convergence characteristics, near-optimal values are achieved in a small number of iterations.

Another significant feature that can be noted from the above simulation result is the robustness of the algorithm for repeated trials. The statistical analysis carried out for multiple independent trials of the algorithm demonstrates that there is a relatively low variance in the objective function values. This demonstrates that the algorithm is not too sensitive to initializations and is capable of producing consistent solutions of high quality. This is a significant feature for OPF problems since they are typically of high dimensionality and are subject to equality and inequality constraints that are nonlinear in nature. The consistent performance of the algorithm demonstrates that it is maintaining a good balance between exploration and exploitation of the search space.

The scalability of the suggested method was also examined by utilizing the IEEE-118 bus test system, which portrays a power network of considerable size with substantially more variables and operational constraints compared to other power systems of lower sizes. The results show that the algorithm retains its stable convergence characteristics even for this large-scale OPF problem. The capacity of the algorithm to deal with this expanded dimension of the search space points to the fact that it can handle power system optimization problems of considerable complexity. This is especially true for modern power systems that are characterized by the presence of numerous power networks and the integration of renewable energy sources.

Although promising results are obtained as demonstrated within this study, some limitations are noted. Like most population-based metaheuristic optimization techniques, this algorithm necessitates repetitive objective function evaluations. This could result in higher computational time for large-scale systems. Moreover, although this algorithm has demonstrated promising results on the test systems, the results could be affected by some characteristics of the OPF problem, such as network size, number of control variables, or constraint level. Therefore, future research could be directed at improving the efficiency of this algorithm using hybrid optimization techniques or parallel search mechanisms.

The overall findings of the presented work indicate that the suggested optimization method is a reliable solution for the OPF problems. The robust convergence characteristic of the suggested method, along with the statistically proven performance improvements, indicates the potential applicability of the suggested method for power system operation.

## Conclusions

In order to achieve an economic operation of a modern power system, system operators use OPF solutions in operation, control and future expansion of system planning. The research paper presents the Jaya optimization tool as a revolutionary solution to overcome OPF problems for maintaining grid stability through improving the voltage profile of load buses and efficient loss reduction of the power system. The Jaya method has been used on IEEE 30-bus, IEEE-57-bus, and IEEE 118-bus test systems to assess its effectiveness and superiority. The numerical results achieved by the Jaya algorithm were compared with recent competitive global optimization methods. The results were particularly noteworthy as all inequality constraints were fully satisfied during the optimization process. These bounds include outputs of active and reactive power generation, thermal limits of lines, voltage magnitudes of load buses, and line loading. The research demonstrates that Jaya achieves higher accuracy and efficiency in most cases in comparison to established algorithms, TLBO, DE, and PSO, with evidence from modern literature investigations. We further acknowledge that the weighted sum method (WSM), while simple and widely used, may not always capture these trade-offs effectively—especially when objectives conflict. Future research will explore Pareto-based multi-objective optimization methods to more accurately represent trade-offs inherent to OPF problems.

## Data Availability

The datasets used and/or analysed during the current study are available from the first author on reasonable request.

## Appendix

See Tables 15 and 16.

**Table 15:** The Jaya algorithm optimum values of control variables of Case 1–Case 6 for the IEEE 30-bus system.

S. no	Control variable (pu)	Case 1	Case 2	Case 3	Case 4	Case 5	Case 6
1	P_G2_	0.4873	0.2	0.4872	0.4811	0.800	0.6633
2	P_G5_	0.2134	0.15	0.214	0.214	0.500	0.5
3	P_G8_	0.208	0.1056	0.2105	0.2416	0.350	0.35
4	P_G11_	0.1186	0.1438	0.1179	0.1279	0.300	0.3
5	P_G13_	0.12	0.12	0.12	0.12	0.400	0.4
6	V_G1_	1.0929	0.995	1.008	1.020	1.0616	1.0737
7	V_G2_	1.0728	0.95	1.057	1.021	1.0577	1.0677
8	V_G5_	1.044	1.0668	1.0329	1.092	1.0381	1.0477
9	V_G8_	1.0499	1.0104	1.0104	1.081	1.0495	1.0539
10	V_G11_	1.0991	1.037	1.039	1.1	1.1	1.1
11	V_G13_	1.05828	1.067	1.0109	1.1	1.07	1.0613
12	T_6−9_	1.1	1.0533	0.914	0.914	1.0858	1.0445
13	T_6−10_	0.9031	0.9	0.9907	0.9747	0.9001	0.9518
14	T_4−12_	0.9757	1.1	0.9549	0.9549	0.9977	0.9928
15	T_28−27_	0.9814	0.967	0.9246	0.9256	0.9772	0.9761
16	Q_Sh10_	0.0434	0.0495	0.05	0.05	0	0.0293
17	Q_Sh12_	0.0447	0.05	0.05	0.05	0.0479	0.05
18	Q_Sh15_	0.0451	0.05	0.05	0.05	0.039	0.0448
19	Q_Sh17_	0.05	0.0021	0.05	0.05	0.0499	0.05
20	Q_Sh20_	0.042	0.0499	0.05	0.05	0.0413	0.0413
21	Q_Sh21_	0.05	0.0482	0.05	0.05	0.05	0.05
22	Q_Sh23_	0.0328	0.05	0.05	0.05	0.0371	0.0332
23	Q_Sh24_	0.05	0.05	0.05	0.05	0.05	0.05
24	Q_Sh29_	0.0284	0.0272	0.05	0.05	0.0257	0.0236
Fuel cost($/h)		799.9676	841.4409	810.2319	818.5353	967.683	942.3443
TVD (p.u)		1.0988	0.0893	0.6223	0.7439	1.0361	1.1261
Emission (ton/h)		0.3358	0.4460	0.3408	0.3375	0.2066	0.2037
Power loss (M.W)		8.9137	15.5359	11.9651	14.145	3.1086	3.2162
L-Index		0.1286	0.1402	0.1283	0.1362	0.1302	0.1286

**Table 16:** The Jaya algorithm optimum values of control variables of case 7- case 10 for the IEEE 57-bus system.

S. no	Control variable	Case-7	Case-8	Case-9	Case-10
1	P_G2_	0.8980	0.9361	0.9999	0.3000
2	P_G3_	0.4531	0.4446	0.4470	1.3158
3	P_G6_	0.7358	0.5873	0.6755	0.9999
4	P_G8_	4.6019	4.6159	4.5949	3.0854
5	P_G9_	0.9584	0.9949	0.9319	0.9986
6	P_G12_	3.5763	3.6563	3.5914	4.0997
7	V_G1_	1.0671	1.0145	1.0670	1.0731
8	V_G2_	1.0718	1.0190	1.0708	1.0740
9	V_G3_	1.0591	1.0075	1.0580	1.0674
10	V_G6_	1.0609	1.0225	1.0601	1.0622
11	V_G8_	1.0736	1.0438	1.0717	1.0695
12	V_G9_	1.0668	1.0228	1.0551	1.0625
13	V_G12_	1.0539	1.0142	1.0509	1.0592
14	T_4−18_	1.0419	1.0998	0.9629	1.0952
15	T_4−18_	0.9440	0.900	0.9738	0.9075
16	T_21−20_	1.0200	0.9746	1.0077	1.0139
17	T_24−25_	0.9534	1.0841	1.0850	1.0318
18	T_24−25_	1.1000	1.1000	1.0999	1.0100
19	T_24−26_	1.0296	1.0040	1.0311	1.0188
20	T_7−29_	0.9968	1.0316	0.9980	0.9961
21	T_34−32_	0.9617	0.9201	0.9526	0.9632
22	T_11−41_	0.9000	0.9000	0.9000	0.9043
23	T_15−45_	0.9866	0.9301	0.9853	0.9925
24	T_14−46_	0.9686	0.9754	0.9670	0.9728
25	T_10−51_	0.9845	0.9971	0.9784	0.9868
26	T_13−49_	0.9458	0.9000	0.9387	0.9470
27	T_11−43_	1.0072	0.9716	0.9907	0.9972
28	T_40−56_	1.0152	0.9950	1.0003	1.0100
29	T_39−57_	0.9546	0.9000	0.9647	0.9694
30	T_9−55_	1.0080	1.0255	1.0025	0.9986
31	Q_sh18_	0.0598	0.0020	0.0029	0.0014
32	Q_sh25_	0.1599	0.2457	0.2697	0.1554
33	Q_sh53_	0.1109	0.3000	0.1360	0.1316
Fuel cost ($\h)		41,657.3500	41,755.6700	41,665.3500	4441.4980
TVD (p.u)		1.7122	0.5889	1.8417	1.7192
L-Index		0.2327	0.2348	0.22	0.2348
Power loss (M.W)		14.7339	16.6558	14.801	9.7924

## List of symbols

U: State/dependent variable
X: Control variable
M (U, X): Objective function
g (U, X): Equality constraint
h (U, X): Inequality constraint
$\:{\mathrm{P}}_{\mathrm{g}\mathrm{i}}$and$\:{\mathrm{Q}}_{\mathrm{g}\mathrm{i}}$: are the active and reactive power generations
Nbus: Number of buses
NLB: is the number of load buses
Ntl: is the number of transmission lines
NGN: are the numbers of generators, and
NC: numbers of VAR compensation units
NTR: numbers of regulating transformers
$\:{\mathrm{P}}_{\mathrm{d}\mathrm{i}}$and$\:{\mathrm{Q}}_{\mathrm{d}\mathrm{i}}$: are the active and reactive load demand
$\:{\mathrm{P}}_{\mathrm{g}\mathrm{i}}$and$\:{\mathrm{Q}}_{\mathrm{g}\mathrm{i}}$: are the active and reactive power generations
$\:{\mathrm{P}}_{\mathrm{L}\mathrm{o}\mathrm{s}\mathrm{s}}$and$\:{\mathrm{Q}}_{\mathrm{L}\mathrm{o}\mathrm{s}\mathrm{s}}$: are the total real and reactive power loss
$\:{\mathrm{V}}_{{\mathrm{g}}_{\mathrm{k}}}^{\mathrm{m}\mathrm{a}\mathrm{x}}$and$\:{\mathrm{V}}_{{\mathrm{g}}_{\mathrm{k}}}^{\mathrm{m}\mathrm{i}\mathrm{n}}$: are the maximum and minimum bus voltage limit of the kth generator bus
$\:{\mathrm{Q}}_{{\mathrm{g}}_{\mathrm{k}}}^{\mathrm{m}\mathrm{a}\mathrm{x}}$and$\:{\mathrm{Q}}_{{\mathrm{g}}_{\mathrm{k}}}^{\mathrm{m}\mathrm{i}\mathrm{n}}$: are the maximum and minimum limits of the reactive power output of kth generator bus
$\:{\mathrm{P}}_{{\mathrm{g}}_{\mathrm{k}}}^{\mathrm{m}\mathrm{a}\mathrm{x}}$and$\:{\mathrm{P}}_{{\mathrm{g}}_{\mathrm{k}}}^{\mathrm{m}\mathrm{i}\mathrm{n}}$: are the maximum and minimum active power limit of the kth generating units
$\:{\mathrm{T}}_{\mathrm{k}}^{\mathrm{m}\mathrm{i}\mathrm{n}}$and$\:{\mathrm{T}}_{\mathrm{k}}^{\mathrm{m}\mathrm{a}\mathrm{x}}$: are the minimum and maximum tap settings of kth transformer
$\:{\:\mathrm{V}}_{{\mathrm{L}}_{\mathrm{k}}}^{\mathrm{m}\mathrm{i}\mathrm{n}}$and$\:{\mathrm{V}}_{{\mathrm{L}}_{\mathrm{k}}}^{\mathrm{m}\mathrm{a}\mathrm{x}}$: are minimum and maximum voltage limits of kth load bus
$\:{\mathrm{S}}_{{\mathrm{l}}_{\mathrm{k}}}^{\mathrm{m}\mathrm{a}\mathrm{x}}$: is maximum MVA flow in kth transmission line
$\:{\:\mathrm{P}}_{1}$,$\:{\:\mathrm{P}}_{2}$, $\:{\mathrm{P}}_{3}$ and$\:{\mathrm{P}}_{4}$: are the penalty factors corresponding to limit violations
$\:{\mathrm{A}}_{\mathrm{i}},{\:\mathrm{B}}_{\mathrm{i}}$, and$\:{\mathrm{C}}_{\mathrm{i}}$: are fuel cost coefficients of the ith generator units
PD: Real load demand
NB: Total number of buses
$\:{\:{\upalpha\:}}_{\mathrm{i}}$,$\:{{\upbeta\:}}_{\mathrm{i}}$,$\:{{\upgamma\:}}_{\mathrm{i}}$,$\:{{\upxi\:}}_{\mathrm{i}}$ and$\:{{\uplambda\:}}_{\mathrm{i}}$: are the emission coefficients of the ith unit

## Abbreviations

MGBICA: Multi-objective Gaussian bare-bones imperialist competitive algorithm
MDE: Modified differential evolution
MFO: Moth flame optimization
FPA: Flower pollination algorithm
ARCBBO: Adaptive real-coded biogeography-based optimization
RCBBO: Real-coded biogeography-based optimization
QOJA: Quasi-oppositional-based Jaya algorithm
IMFO: Improved moth-flame optimization
GWO: Grey wolves optimization
GBICA: Gaussian bare-bones imperialist competitive algorithm
MPSO: Modified Particle Swarm Optimization
ABC: Artificial bee colony algorithm
SKH: Study the krill herd algorithm
ECHT-DE: Ensemble of constraint handling techniques-differential evolution
SF-DE: Superiority of feasible solutions-differential evolution
SP-DE: Self-adaptive penalty-differential evolution
MGOA: Modified grasshopper optimization algorithm
GOA: Grasshopper optimization algorithm
